# Improved microcanonical instanton theory

**DOI:** 10.1039/d2fd00063f

**Published:** 2022-08-05

**Authors:** Joseph E. Lawrence, Jeremy O. Richardson

**Affiliations:** Laboratory of Physical Chemistry, ETH Zürich 8093 Switzerland jlawrence@ethz.ch

## Abstract

Canonical (thermal) instanton theory is now routinely applicable to complex gas-phase reactions and allows for the accurate description of tunnelling in highly non-separable systems. Microcanonical instanton theory is by contrast far less well established. Here, we demonstrate that the best established microcanonical theory [S. Chapman, B. C. Garrett and W. H. Miller, *J. Chem. Phys.*, 1975, **63**, 2710–2716], fails to accurately describe the deep-tunnelling regime for systems where the frequencies of the orthogonal modes change rapidly along the instanton path. By taking a first principles approach to the derivation of microcanonical instanton theory, we obtain an improved method, which accurately recovers the thermal instanton rate when integrated over energy. The resulting theory also correctly recovers the separable limit and can be thought of as an instanton generalisation of Rice–Ramsperger–Kassel–Marcus (RRKM) theory. When combined with the density-of-states approach [W. Fang, P. Winter and J. O. Richardson, *J. Chem. Theory Comput.*, 2021, **17**, 40–55], this new method can be straightforwardly applied to real molecular systems.

## Abstract 2

We develop a microcanonical version of instanton theory for studying deep tunnelling reactions under the statistical assumptions of RRKM. The new theory provides a correction to the theory of Chapman, Garrett and Miller for non-separable systems.

## Graphical abstract

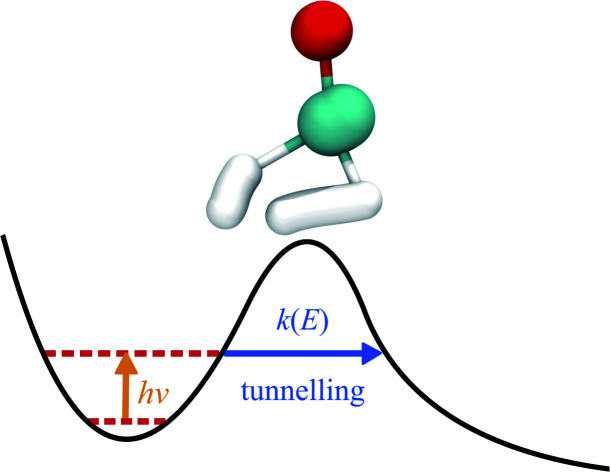

## 1 Introduction

Instanton theory has now become a well-established method for the calculation of thermal reaction rates and can be routinely applied to treat systems in full molecular detail using state-of-the-art electronic-structure theory.^1–10^ By finding the optimal semiclassical tunnelling pathway on the potential-energy surface, instanton theory generalises Eyring transition-state theory to include the effect of tunnelling.^11–13^ Unlike simple one-dimensional tunnelling corrections,^14–19^ instanton theory is equally applicable to both separable and highly non-separable systems, where the tunnelling path may deviate significantly from the minimum-energy path. And, as it makes no assumptions about the form of the potential, it gives an approach which can be applied to deep tunnelling, where perturbative corrections at the transition state fail.^19,20^

For certain reactions, however, assuming thermal equilibrium is not valid. An important example is unimolecular dissociation initiated by photoexcitation at a specific energy,^21,22^ but more generally, this can occur for any multistep reaction where there is insufficient time to thermalise between successive steps.^23–27^ In such cases, one must use a microcanonical rather than canonical approach to reaction rates. The cornerstone of microcanonical rate theory is the Rice–Ramsperger–Kassel–Marcus (RRKM) theory,^28–30^ which generalises Eyring transition-state theory for systems in the microcanonical ensemble. Like Eyring transition-state theory, RRKM does not include the effects of tunnelling. And, whilst there exist simple one-dimensional tunnelling corrections to RRKM,^17^ there does not yet exist a well-established microcanonical version of instanton theory capable of accurately capturing the influence of tunnelling in both separable and non-separable systems.

In order to discuss the derivation of both thermal and microcanonical instanton theory, it will be helpful to make use of some ideas from asymptotic analysis.^31^ As such, we give here a brief overview of the key concepts used later. Firstly, the statement that two functions, 
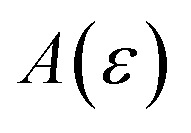
 and 
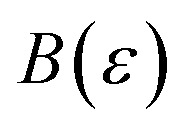
 are asymptotic as 
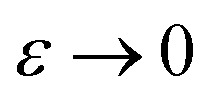
 (written mathematically as 
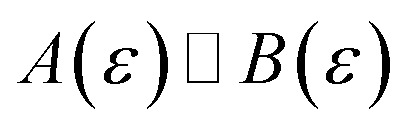
, as 
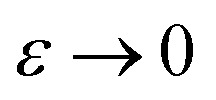
) is equivalent to the statement 
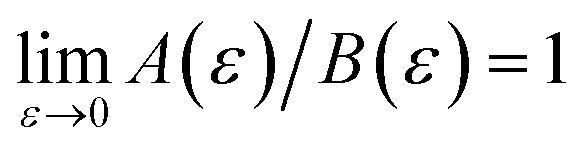
. Importantly, provided *ε* is sufficiently close to zero, then 
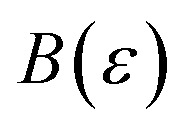
 is expected to provide a reasonable approximation for 
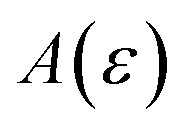
. Of course, such approximations are not unique and only imply that 
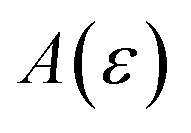
 and 
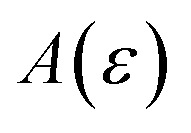
 agree to leading order in *ε*. Asymptotic relations are particularly useful for the approximate evaluation of integrals. In particular, we will make repeated use of steepest descent integration, which uses the asymptotic relation (known as Laplace’s method)1
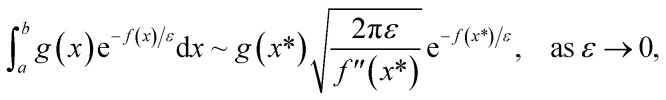
where 
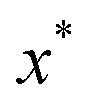
 is the minimum of the (real) function 
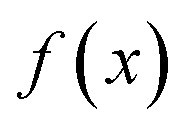
 in the interval 
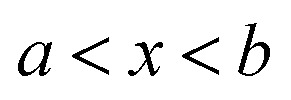
. This can be generalised to approximate the integral of a complex analytic function by first deforming the contour of integration to pass through a saddle point of the function, leading to an integral to which Laplace’s method can be applied. Note that for a finite value of *ε*, the accuracy of this expression will depend on how quickly 
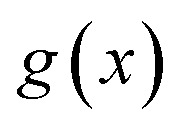
 varies around 
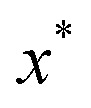
 relative to the width of the peak of 
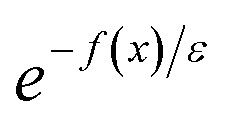
, and also on how well the peak is approximated by a Gaussian. Clearly, the choice of the perturbation parameter will affect the accuracy of the resulting approximation, and, as with all perturbative theories, a physically sensible choice is needed to get accurate results. Often, one employs an asymptotic approximation, known as the semiclassical approximation,^32–34^ where the perturbation parameter is associated with ℏ in the quantum-mechanical propagator, 
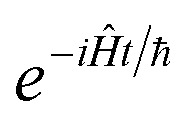
.

For thermal rates, the result of taking the semiclassical limit is instanton theory,^11,34,35^ which has been demonstrated to constitute an accurate approximation for many real molecular systems. Unfortunately, for microcanonical reactions, naively performing integrals by steepest descent 
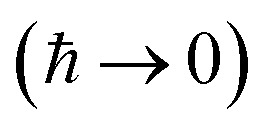
 to give the leading asymptotic term results in a theory which does not recover the correct result for separable multidimensional systems.^11,36^ This led Chapman, Garrett and Miller to suggest a corrected microcanonical instanton theory based on an *ad hoc* “unexpansion” of a Taylor series.^37^ Here, we will show that whilst it recovers the correct result in the separable limit, for non-separable reactions involving large changes in the frequency of orthogonal modes along the instanton path, the approach suggested by Chapman *et al.* breaks down. The aim of this paper is thus to explore how one can derive a microcanonical instanton theory which retains the desirable properties of Chapman *et al.*’s method, but which fixes the issues found in highly non-separable systems. Section 2 reviews the fundamentals of microcanonical rate theory, as well as thermal instanton theory. Section 3 first discusses previously proposed microcanonical instanton theories, explaining their issues, and then goes on to derive an improved microcanonical instanton method. Section 4 applies the method to a simple (but realistic) unimolecular dissociation reaction and Section 5 concludes.

## 2 Background theory

The (canonical) thermal rate constant, 
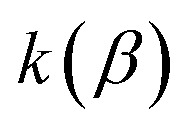
, can be written in terms of an integral over the cumulative reaction probability as^38,39^2
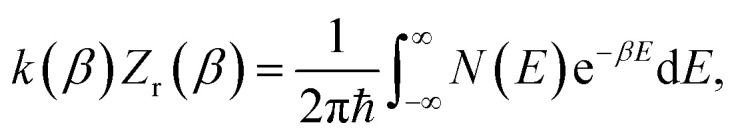
where 
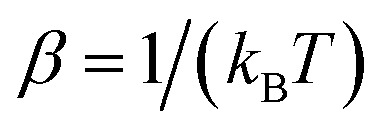
 is the inverse temperature, 
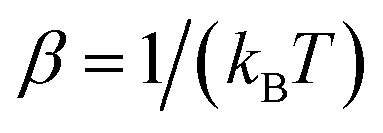
 is the reactant partition function, and 
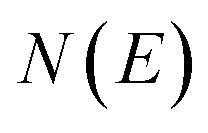
 is the cumulative reaction probability at energy 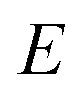 (which can be thought of as the microcanonical number of reactive states). The cumulative reaction probability can be related to the microcanonical rate constant, 
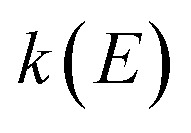
, according to the expression3
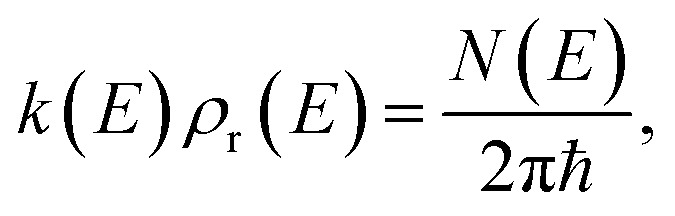
where 
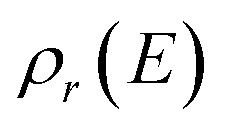
 is the reactant density of states, which satisfies4
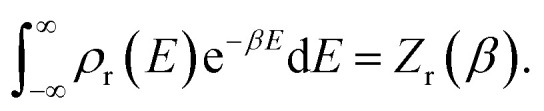


Microcanonical rate constants generalise the concept of thermal rates by replacing the assumption that the system is in thermal equilibrium with the assumption that the system is in microcanonical equilibrium, *i.e.* that all reactant states of energy 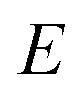 are equally likely to be populated.^28–30^

### 2.1 RRKM and transition-state theory

The traditional starting point for the understanding of microcanonical rates is the Rice–Ramsperger–Kassel–Marcus (RRKM) theory. In addition to the central assumption of equally populated reactant states, RRKM assumes that motion along the reaction coordinate can be separated from orthogonal degrees of freedom, and that these orthogonal degrees of freedom can be treated with a rigid rotor and harmonic oscillator approximation. In the following and later sections, for notational simplicity, we ignore rotational degrees of freedom and briefly discuss how to include their effects in Section 4.1. For a system with 
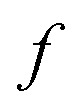
 internal degrees of freedom, the RRKM expression for the cumulative reaction probability can then be written as a sum over the vibrational quantum states at the transition state as5
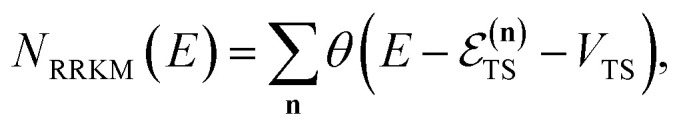
where 
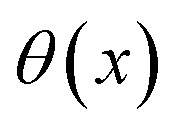
 is the Heaviside step function, 
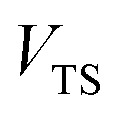
 is the potential energy at the transition state, and6
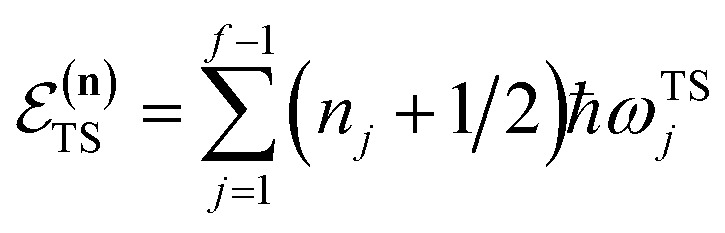
is the vibrational energy of the orthogonal degrees of freedom, with frequencies 
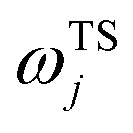
, described by the quantum numbers 
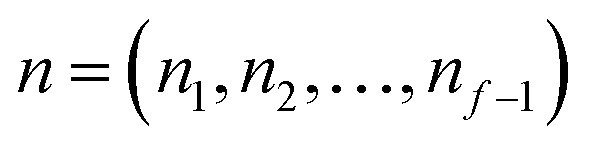
. In order to make the connection to theories that will come later, it is helpful to rewrite the RRKM cumulative reaction probability in terms of an integral over the energy in the reaction coordinate 
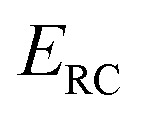
 as7
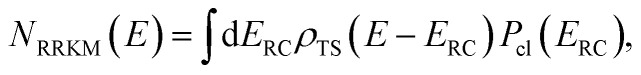
where 
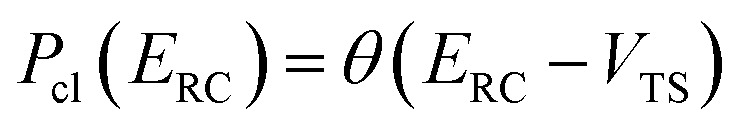
 is the classical transmission probability for a trajectory with energy 
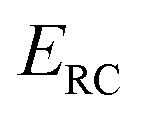
 in the reaction coordinate, and8
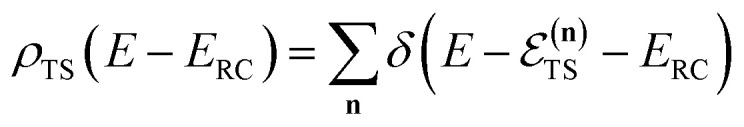
is the density of states at the transition state. As is obvious from the assumptions of RRKM theory, it is closely related to the standard canonical Eyring transition-state theory. And, in fact, simply integrating the RRKM cumulative reaction probability shows that the two are in one-to-one correspondence,9
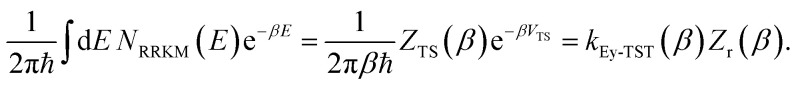


Here, the transition-state partition function is given by the standard expression10
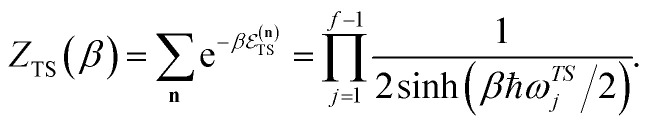


### 2.2 Separable tunnelling corrections to RRKM

Although RRKM includes the effect of the quantum-mechanical zero-point energy on the reaction rates, it does not allow for tunnelling along the reaction coordinate. Within the separable approximation of RRKM theory, tunnelling can be included by replacing the classical transmission probability 
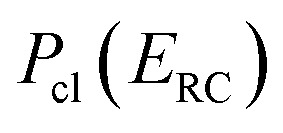
 with a one-dimensional tunnelling probability,^17^ to give11
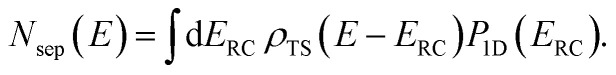


Often, this one-dimensional tunnelling probability is chosen to be the exact one-dimensional transmission probability for an asymmetric Eckart barrier matched to give the same reactant energy, 
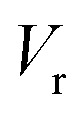
, product energy, 
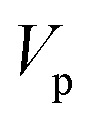
, transition-state energy, 
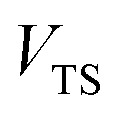
, and barrier frequency, 
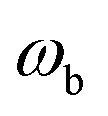
, as the physical problem.^17^ Alternatively, and more relevant for what will follow, one can instead use a semiclassical approximation to the one-dimensional barrier transmission probability. At energies below the barrier top 
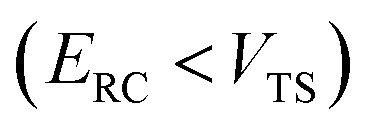
, the Wentzel–Kramers–Brillouin (WKB) approximation gives the transmission probability as^18^12
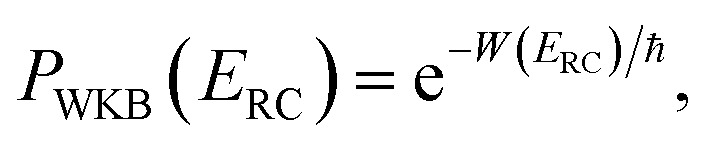
where 
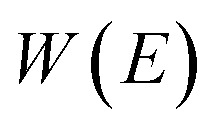
 is the reduced action13
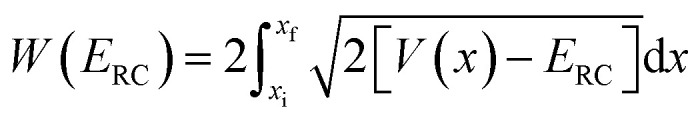
for the one-dimensional potential 
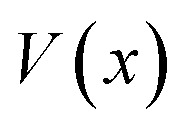
 along the mass-weighted reaction coordinate, 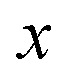. Note that 
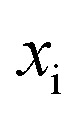
 and 
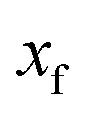
 are the turning points of a trajectory on the upturned potential, satisfying 
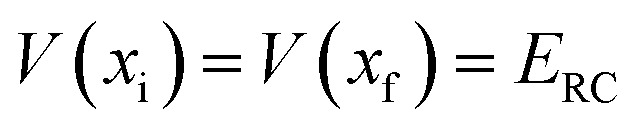
. Noting that the exact transmission probability for a parabolic barrier can be written as 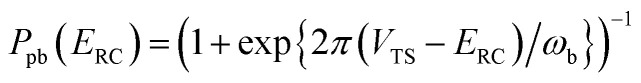, an alternative to the simple WKB formula has been proposed,^18^ which can more accurately capture the transmission probability near the barrier top. The modified formula14
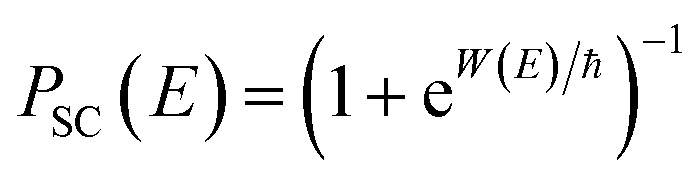
takes the same form as the parabolic barrier transmission probability, with the reduced action defined as15
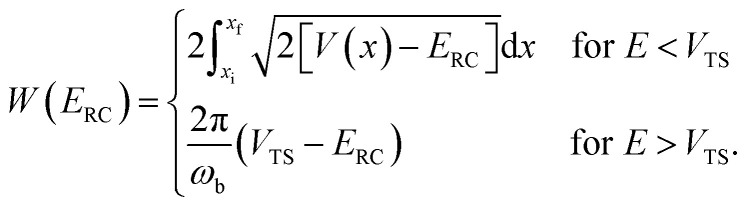


We can also thermalise the resulting separable tunnelling-corrected microcanonical rate to give16
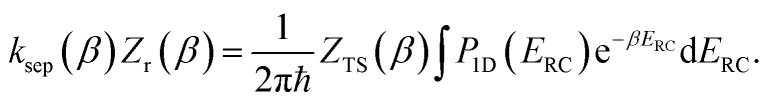


In the low-temperature, deep-tunnelling regime, this can be evaluated by steepest descent integration using 
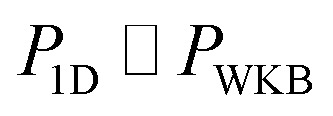
 to give17
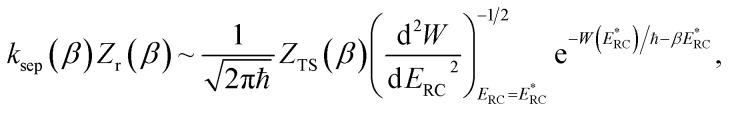
where 
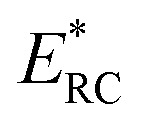
 satisfies the steepest descent condition18
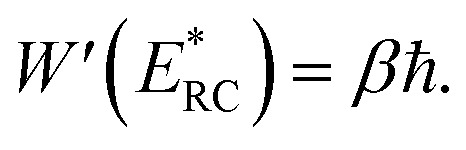


Unfortunately, the separable approximation is known to be inaccurate for many real chemical systems. This often occurs in systems where light atoms tunnel through a high but narrow barrier, avoiding the classical transition state, in a process known as corner cutting.^37,40,41^ This can mean the orthogonal frequencies along the true tunnelling path differ significantly from those at the classical transition state. More generally, the separable approximation will break down for any system in which the frequencies along the tunnelling pathway change significantly with energy.

### 2.3 Thermal instanton theory

For thermal rates, instanton theory provides a rigorously justified and well tested approach to go beyond the separable approximation. Thermal instanton theory can be derived as a rigorous semiclassical 
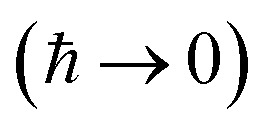
 limit of the thermal flux correlation formalism for the rate.^34^ The resulting instanton approximation to the rate (valid in the “deep-tunnelling” regime) is given by^11,34,35,42^19
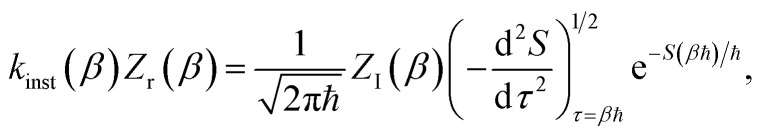
where 
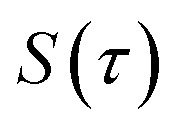
 is the action for the instanton trajectory. Formally, the instanton trajectory is an imaginary-time periodic orbit under the reaction barrier, with total period *τ* = *β*ℏ. The periodic orbit corresponds to a stationary value of the action functional,20
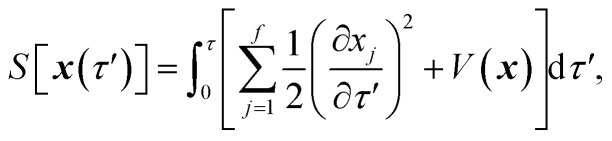
which is typically practically found by discretising the instanton trajectory according to 
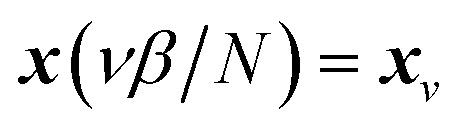
. The approximate instanton trajectory is then given by a first-order saddle point of the discretised action,^12,13^21
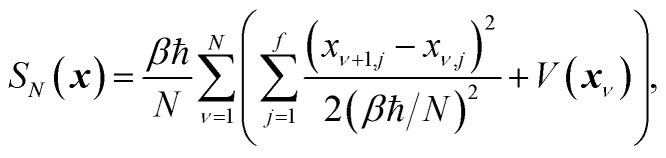
with the approximation becoming more and more accurate as 
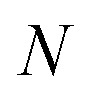
 increases.

To make the connection with the separable approximation in the previous subsection, and to aid the discussion of the microcanonical versions of instanton theory that will follow, we can transform the instanton rate expression into an equivalent form by making use of a series of standard identities from Lagrangian mechanics.^33^ Firstly, the total derivative of the action with respect to imaginary time for a classical periodic orbit is just the energy of the corresponding orbit22
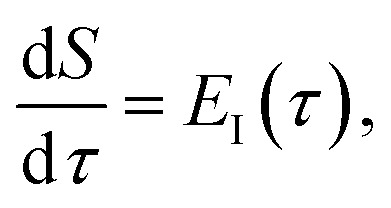
which we refer to as the instanton energy, 
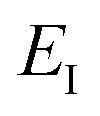
. Instead of the action as a function of the period of the orbit, we can instead perform a Legendre transformation to consider the reduced action as a function of the instanton energy23
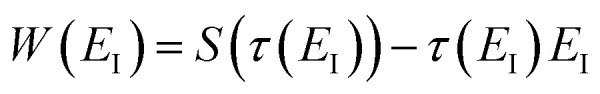


Using the chain rule, one can show that24
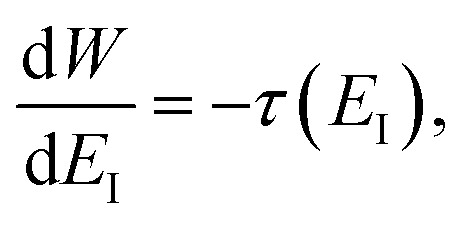
and25
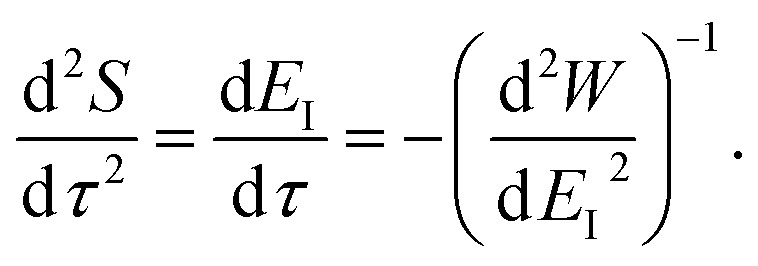


By combining these identities, we can then see that the thermal instanton rate takes the same form as the separable rate in eqn (17),26
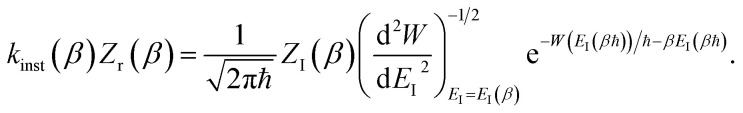


There are, however, two main differences between eqn (26) and (17). Firstly, in eqn (26), the reduced action is calculated along the instanton path, which may not pass through the classical transition state. And secondly, the transition-state partition function is replaced by the instanton partition function27
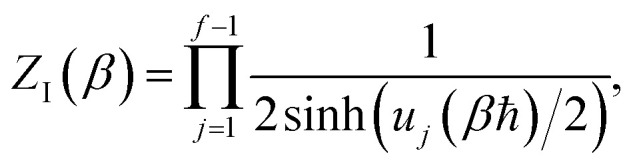
where 
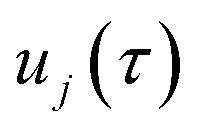
 are the classical stability parameters for the trajectory.^11^ A detailed discussion of the stability parameters, including how they can be efficiently calculated, is given for completeness in the Appendix. For now, it is sufficient to note that in the case that the problem is truly separable, the stability parameters are equal to the period multiplied by the transition-state frequency, 
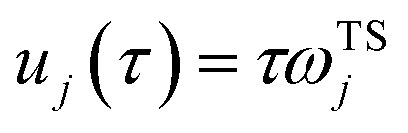
, and, hence, we can think of the stability parameters in terms of effective *τ*-dependent frequencies, 
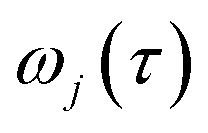
, by defining28
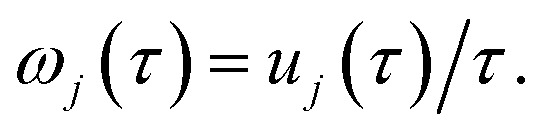


## 3 Microcanonical instanton theory

Given the success of instanton theory for thermal problems, it seems natural to ask, does there exist a microcanonical version of instanton theory? It is of historical interest that the original derivation of the thermal instanton from scattering theory by Miller proceeded *via* a microcanonical expression for the rate.^11^ However, although the final thermal instanton expression Miller derived is typically very accurate for many chemical systems, the microcanonical theory that it was derived from has a number of issues. Here, we take a first principles approach to the derivation of a microcanonical instanton theory. We begin by summarising previous attempts to derive a microcanonical instanton theory, analysing their associated issues, before suggesting some improved expressions.

In order to illustrate the accuracy and deficiencies associated with each of the methods, we will make use of a simple two-dimensional generalisation of the Eckart barrier model, described by the potential29
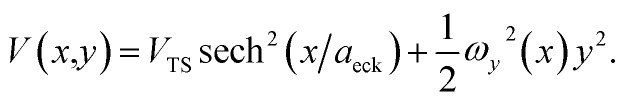


We consider two different functional forms for the frequency of the *y* coordinate,30
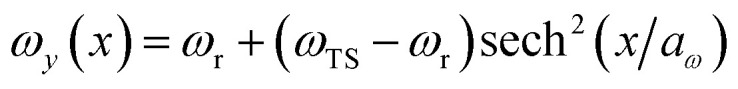
which we will refer to as model 1, and31
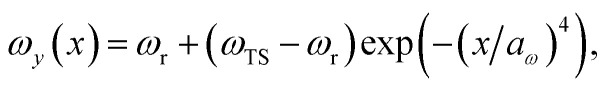
which we will refer to as model 2. Note that this generalises the model studied in ref. 36, which employed a constant frequency, *ω*_*y*_. In all cases, we work in mass-weighted coordinates, such that *m* = 1 for both degrees of freedom, and in units where ℏ = 1.

### 3.1 Deriving a microcanonical instanton from first principles

A natural starting point when trying to derive microcanonical instanton theory is the exact quantum expression for the cumulative reaction probability. For a system obeying scattering boundary conditions, the cumulative reaction probability can be written in terms of the reactive flux operator and the imaginary part of the Green’s function as^39,43^32
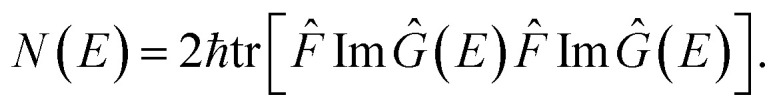


The flux operator is defined as33
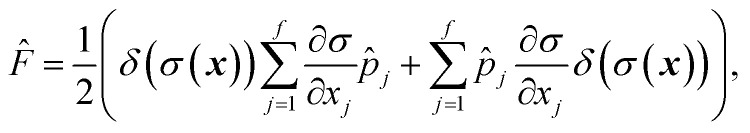
where 
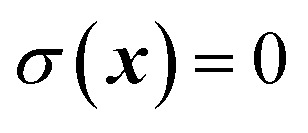
 is a dividing surface which separates reactants 
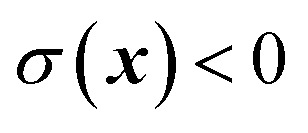
 from products 
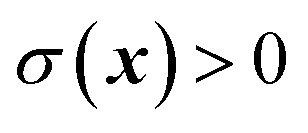
. And the imaginary part of Green’s function can be written as34
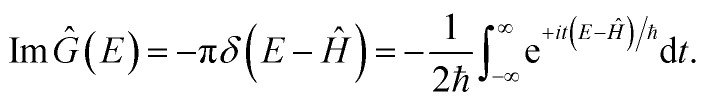


Previous efforts to derive a microcanonical instanton theory from first principles have begun by obtaining a semiclassical expression for the Green’s function, replacing the quantum-mechanical propagator with the van Vleck propagator and integrating over time by steepest descent.^36^ Then, by integrating over the remaining coordinates by steepest descent 
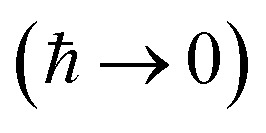
, one obtains the original microcanonical theory derived by Miller.

A better understanding of the approximations involved in this derivation can be gained by noting that the order of integration does not matter. On this basis, we can begin by rewriting the cumulative reaction probability explicitly in terms of the two time integrals as35
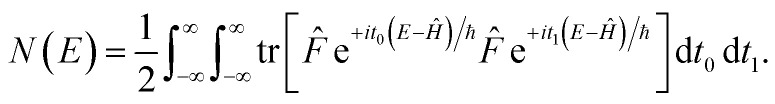


Then, we can make a variable transformation in the two time coordinates to 
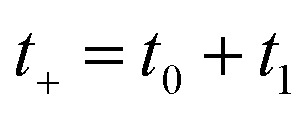
 and 
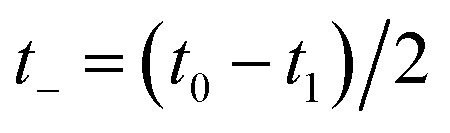
, such that 
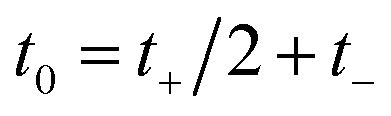
 and 
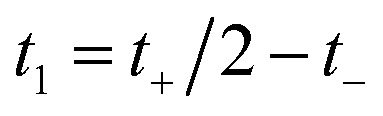
. This allows us to write the cumulative reaction probability as36
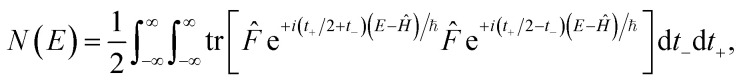
or equivalently37
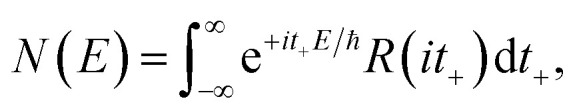
where (defining *τ*_+_ ≡ *it*_+_)38
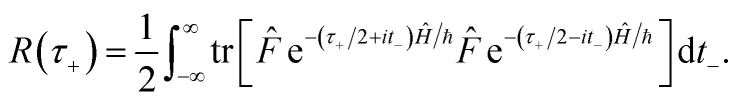


Eqn (37) is simply the Bromwich integral for the inverse Laplace transform of the thermal rate (at temperature *β* = *τ*_+_/ℏ) written in terms of the flux–flux autocorrelation function (eqn (38)). By deriving these transforms step-by-step, we wish to illustrate that the results of working with the Green’s function formalism or taking the inverse Laplace transform of the thermal rate are necessarily equivalent. This equivalence remains true even under the semiclassical approximation, since (done consistently) the order of performing steepest descent integrals does not matter. Thus, before analysing Miller’s original microcanonical theory, we first perform the integrals over position and 
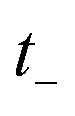
 by steepest descent to give the instanton expression for the thermal rate39
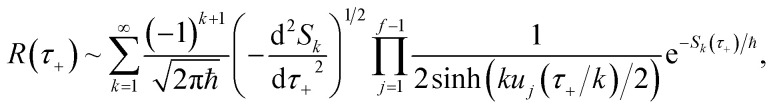
where 
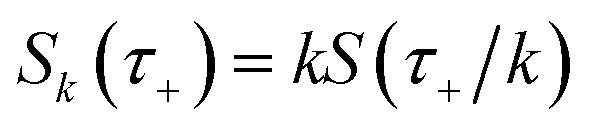
. Only the first term in this series 
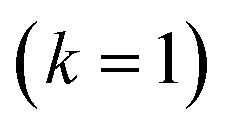
 has been rigorously derived from first principles,^34,35^ however in order to make the connection with Miller’s original microcanonical theory, one must also include these higher order terms following ref. 11.

### 3.2 Miller’s original microcanonical instanton theories

In order to motivate the development of our new microcanonical instanton theory, we begin by discussing the original theory of Miller and the *ad hoc* correction of Chapman *et al.* To arrive at Miller’s original microcanonical instanton theory, one simply combines eqn (39) with eqn (37) and takes the integral over 
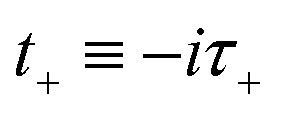
 by steepest descent 
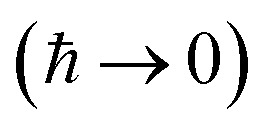
 to give40
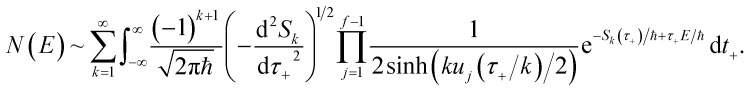


The steepest descent condition is then 
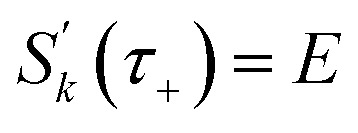
 or, equivalently,41
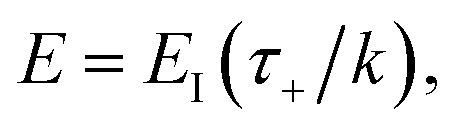
which can be inverted to give 
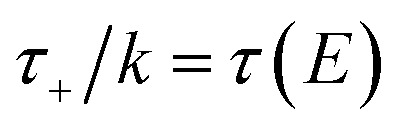
. Using this relation, along with the definition of 
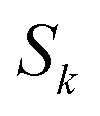
, and noting that the second derivative arising from the steepest descent integration cancels with the existing term, gives42
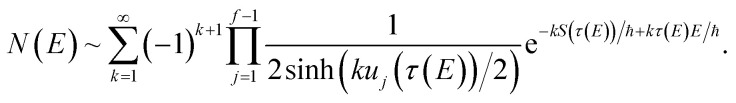


On replacing the action with the reduced action, this immediately simplifies to give Miller’s original microcanonical expression43
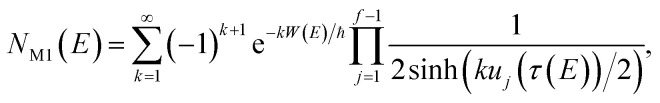
where 
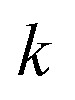
 can be interpreted as the number of times the periodic trajectory orbits.

In the simple one-dimensional case, this recovers the semiclassical result of eqn (14)44
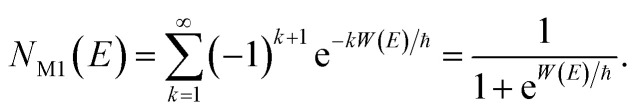


However, as was observed by Miller,^11^ this does not recover the correct result for a separable multidimensional system (eqn (11)). To see this more clearly, one can integrate over 
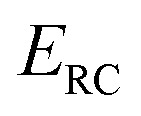
 in eqn (11) using the semiclassical transmission probability from eqn (14) to give45
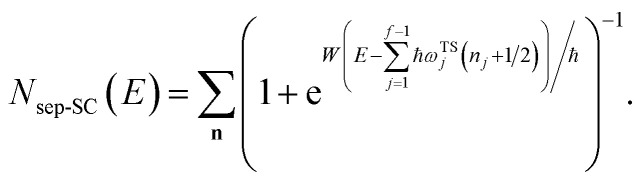


We can then compare this with Miller’s original microcanonical instanton expression (eqn (43)) for a separable system by expanding each instance of 
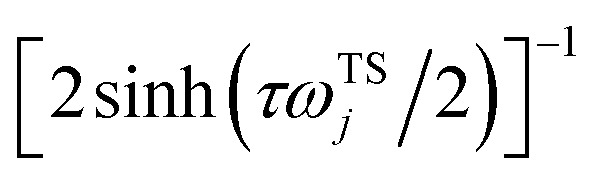
 as a sum over quantum states46
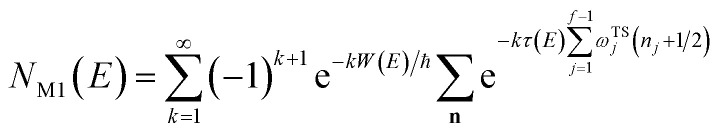
and evaluating the sum over *k* to give47
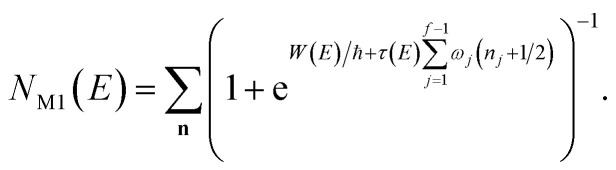


Miller noted that (using eqn (24))48
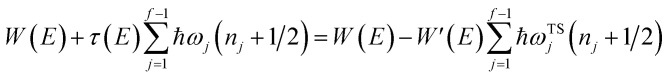
is just the first two terms in a Taylor expansion of 
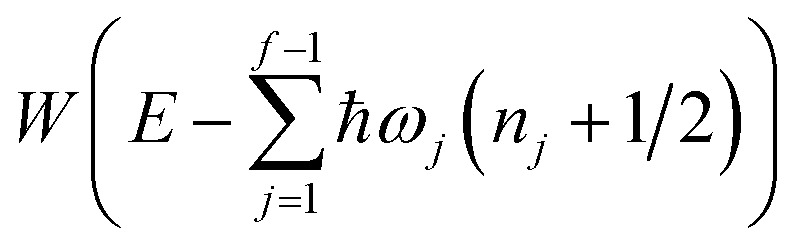
 in powers of ℏ,^11^ and with Chapman and Garrett suggested that the original microcanonical instanton theory (eqn (43)) could be improved by “unexpanding” the Taylor series to give49
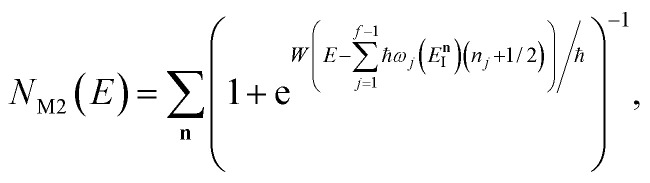
where 
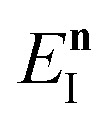
 satisfies the implicit equation 
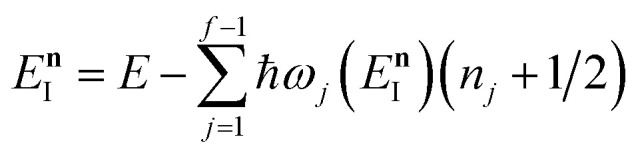
.^37^

**Fig. 1:**
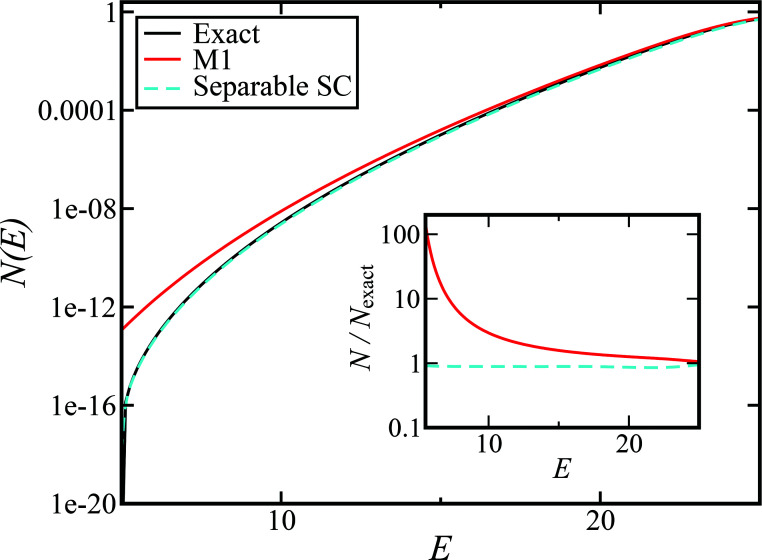
A comparison of the original microcanonical theory (M1) and the separable semiclassical (SC) theory, which is equivalent to the modified instanton theory suggested by Chapman, Garrett and Miller^37^ (M2) for the separable model system used here, with *V*_TS_ = 20, 
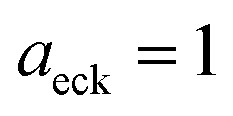
 and *ω*_TS_ = *ω*_r_ = 10. Note that the standard RRKM result (ignoring tunnelling corrections) is not shown as it zero for all relevant energies. The inset shows the relative error.

Fig. 1 illustrates the breakdown of the original microcanonical theory for a separable version of the two-dimensional Eckart model, with 
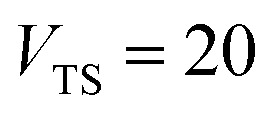
, 
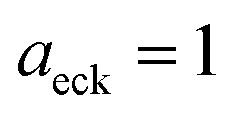
, 
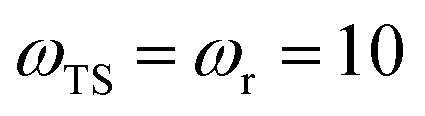
. The breakdown of the theory will clearly be most pronounced for systems where there is a large amount of energy in the non-reactive modes. This figure also clearly illustrates that for this simple system, the separable semiclassical result (and hence also Chapman *et al.*’s method, which is equivalent for this system) very accurately captures the behaviour of the exact cumulative reaction probability. This, however, raises the question, how well does Chapman *et al.*’s *ad hoc* fix do for more general (*i.e.* non-separable) systems?

#### 3.2.1 Density-of-states approach

Before we discuss the accuracy of Chapman *et al.*’s method, we begin by discussing an efficient implementation of the method. Unfortunately, the sum over quantum states and the need to solve the implicit equation for 
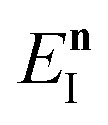
 makes the method, as written above, impractical for many systems of chemical interest. Recently, however, it has been suggested that these difficulties can be avoided by rewriting the cumulative reaction probability in terms of an integral over the instanton density of states.^44^ To achieve this, one begins by introducing a series of delta functions to write50
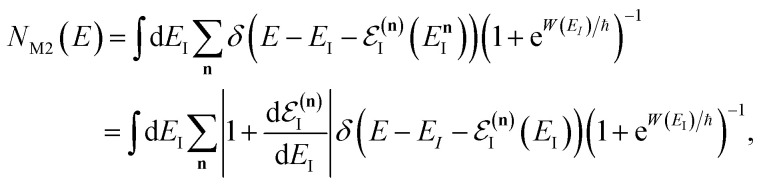
where the vibrational energy is given by51
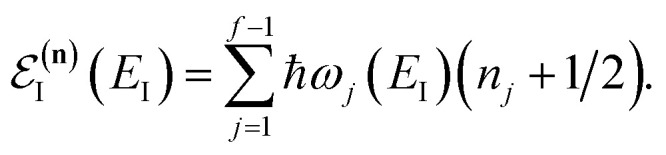


Note that in going from the first to the second line of eqn (50), the implicitly defined 
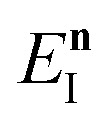
 has been replaced by 
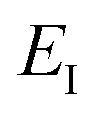
 and an appropriate prefactor introduced using the property of the Dirac delta function: 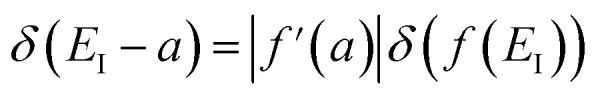 for a function 
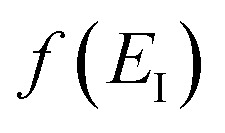
 which has a single root at 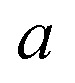. Then, assuming that the vibrational energy changes slowly with 
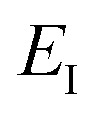
, this prefactor can be dropped to give the microcanonical density-of-states (DoS) instanton^44^52
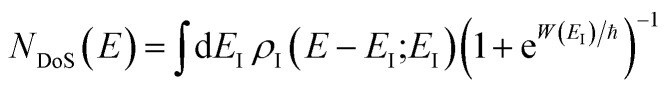
where the instanton density of states is defined as53
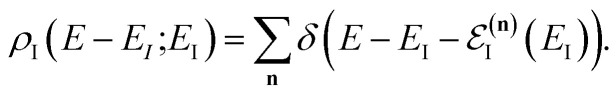


Written in this form, it may seem that one still has to perform a sum over quantum states, which can be impractical for large systems. However, the instanton density of states can be accurately approximated using the stationary-phase approximation to the inverse Laplace transform (SPA-ILT). This is straightforward to evaluate even for large molecular systems,^44^ making this a practical method for real systems. This was demonstrated explicitly for the unimolecular dissociation of a Criegee intermediate,^44^ which gave good agreement with the experimental measurements,^45^ although the reaction was not a particularly challenging test as the separable approximation is in relatively good agreement too. We note in passing that it has been proposed that one can extract microcanonical data from the closely related RPMD rate theory^46^ using similar SPA-ILT methods.^47^

#### 3.2.2 Breakdown for strongly non-separable systems

In order to understand the limitations of both Chapman *et al.*’s microcanonical theory and the DoS method (which builds on it), it is useful to consider the thermal rate that one would obtain upon integrating over the corresponding cumulative reaction probability according to eqn (2). For Chapman *et al.*’s method, this gives54
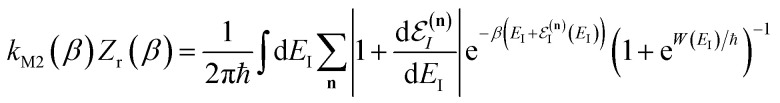
and for the DoS method, in which the sum can be performed analytically, one obtains55
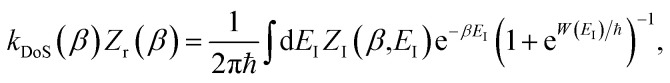
where we have defined an effective instanton partition function which depends separately on the temperature and instanton energy, according to56
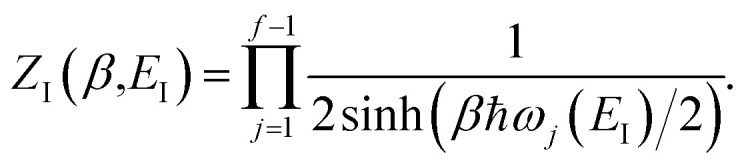


Unfortunately, neither of these expressions necessarily accurately recover the thermal instanton rate. To see this, we can consider integrating over the instanton energy, 
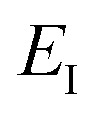
, by steepest descent. In doing so, we see that only when 
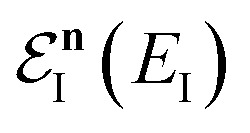
 is slowly varying, such that the DoS method and M2 are equivalent and the instanton partition function, 
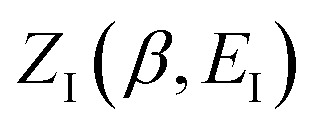
, can be treated as a slowly varying prefactor in the steepest descent integration, will we recover the original thermal instanton rate. However, in many cases with high-frequency modes, the vibrational partition function may be expected to vary strongly with the instanton energy, 
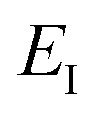
.

**Fig. 2:**
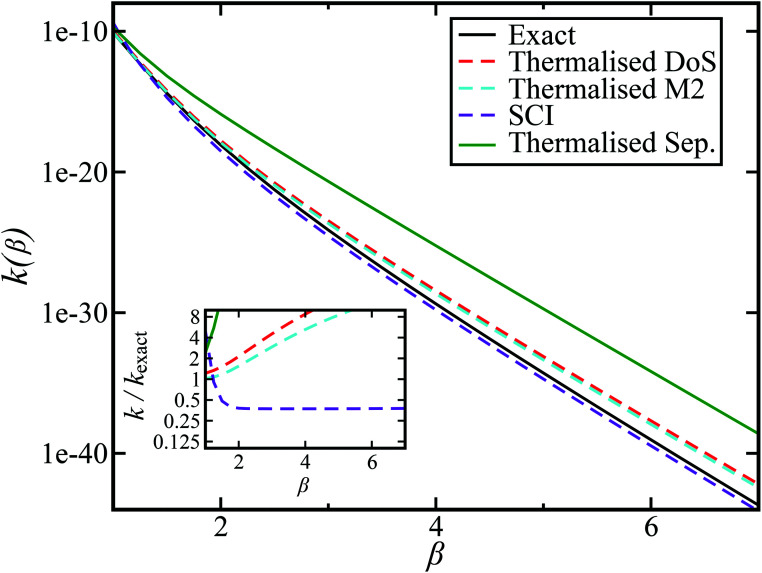
A comparison of the exact thermal rate, the semiclassical instanton rate (eqn (26)) and the thermalised microcanonical rates from Chapman *et al.*’s method (M2) and the DoS method for model 1, with *V*_TS_ = 20, 
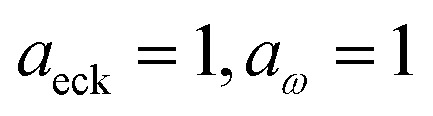
, *ω*_TS_ = 5 and *ω*_r_ = 20. Note that we have also included the separable approximation given by eqn (45), which breaks down very severely for this system. The inset shows the relative error.

Fig. 2 compares the thermal rates calculated using Chapman *et al.*’s method (denoted as M2) and the DoS method with both the thermal semiclassical instanton rate and the exact rate for model 1 with 
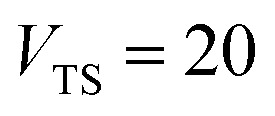
, 
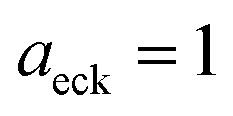
, 
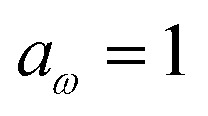
, 
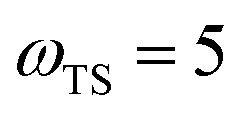
 and 
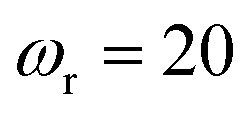
. This model is strongly non-separable due to the large change in the frequency of the *y* coordinate from the reactant asymptote to the transition state. We see that both the thermalised DoS and M2 agree relatively closely, showing that the DoS approximation to M2 is valid for this system. However, neither the DoS method or M2 accurately describes the behaviour of the exact rate except at high temperature. In particular, the thermalised microcanonical instantons do not exhibit the correct slope at low temperature and thus deviate more and more from the exact result. This is in contrast to the semiclassical thermal instanton rate (eqn (26)), which does at least correctly describe the slope of the exact rate at low temperature, where the error is approximately −63% (this remaining error can be attributed to anharmonic effects and will be discussed later in the paper, in Section 3.4). Of course, whilst the thermal instanton is relatively accurate at low temperatures, at the highest temperatures its error grows rapidly due to the well known breakdown of thermal instanton theory near the cross-over temperature (which occurs at *β* ≈ 1 for this system).^36^

### 3.3 Improved microcanonical instanton theories

In the following, we aim to derive an improved microcanonical instanton theory which is able to accurately recover the thermal instanton result when integrated over energy, whilst still recovering the semiclassical tunnelling-corrected RRKM result for separable systems. We note that previously, instanton methods have been proposed which attempt to include the effects of the orthogonal degrees of freedom in an average sense,^36,44^ and hence do not recover the separable limit; as such, these methods will not be considered further in this work. In order to motivate our derivation, we begin by returning to the derivation of Miller’s original microcanonical instanton theory in order to better understand why integrating by steepest descent as ℏ→0 does not recover the desired result for a separable system. Hence, we begin by considering the case of a separable system for which eqn (40) becomes57
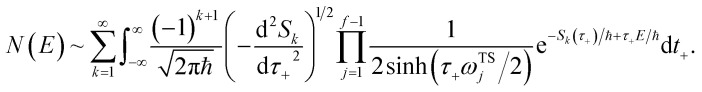


Now, by expanding each of the hyperbolic sine functions as a sum over quantum states, we obtain58
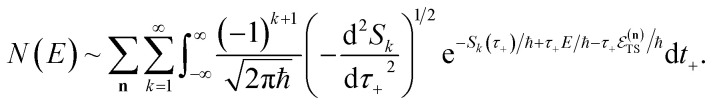


Written in this form, we can see why the semiclassical steepest descent integration 
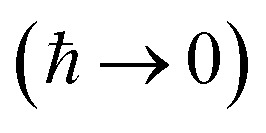
) does not recover the separable semiclassical RRKM result. Unlike in RRKM, where the orthogonal modes are treated separately from the reaction coordinate, here all modes are treated together. This leads the steepest descent approximation to give a poor approximation because, in the ℏ → 0 limit, the vibrational energy of the orthogonal modes, 
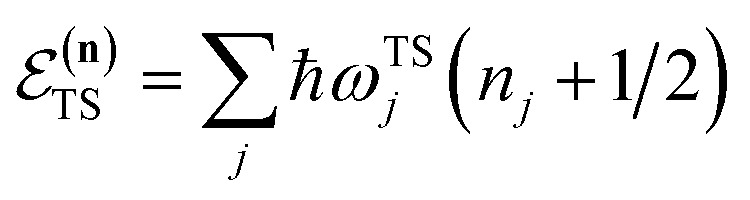
, will go to zero. If we instead hold the value of 
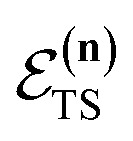
 constant whilst taking the ℏ → 0 limit, then the resulting steepest descent condition 
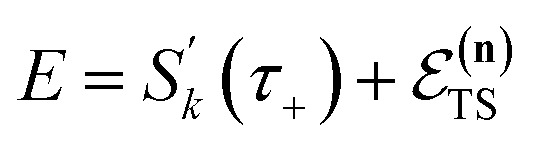
 is equivalent to59
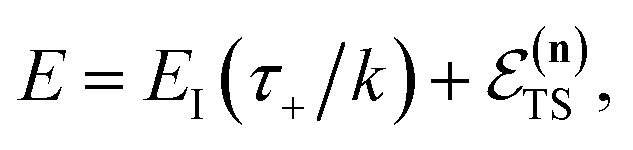
which can be inverted to give 
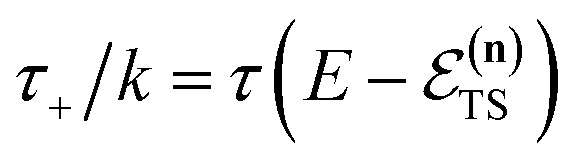
, and the resulting expression for the cumulative reaction probability is60
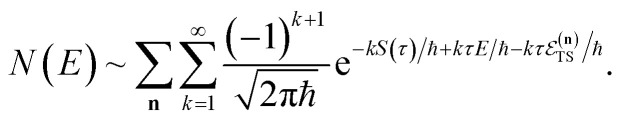


This is equivalent to the desired separable semiclassical tunnelling correction to RRKM given in eqn (11). Hence, we see that to obtain the correct result for a separable system, we must hold the energy (rather than the frequency) of the orthogonal modes fixed whilst taking the ℏ → 0 limit in performing the steepest descent integration over 
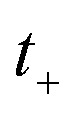
.

#### 3.3.1 An improved method 1: direct sum over effective states

This naturally suggests that one should do the same thing in the non-separable case. As we shall see, the resulting approach has some practical difficulties, and hence an alternative approach that avoids these difficulties will be explored in Section 3.3.2. Following the same procedure as above, one begins by expanding the sinh as a sum over (now effective) quantum states61
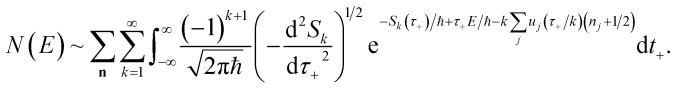


Then, performing the integration by steepest descent whilst fixing the magnitude of 
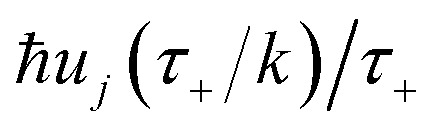
 even as ℏ → 0, results in the steepest descent condition62
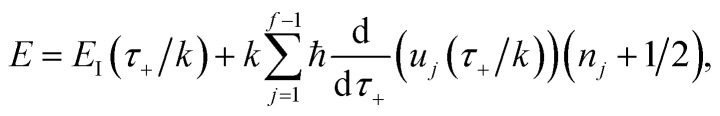
and performing the integral by steepest descent leads to the following expression for the cumulative reaction probability:63
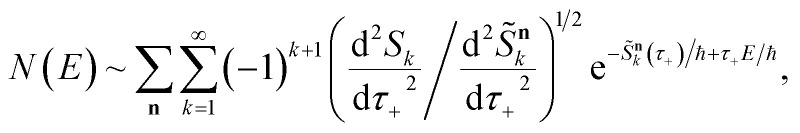
where we have defined a modified action 
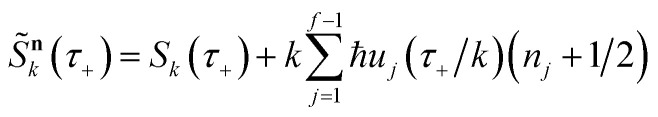
. This can then be simplified by first noting that 
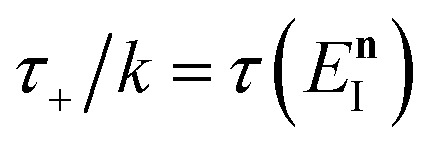
, where 
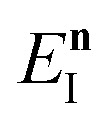
 satisfies the implicit equation64
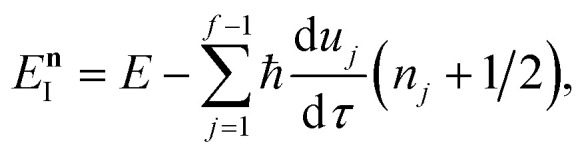
and then by rewriting the modified action in terms of a modified reduced action as65
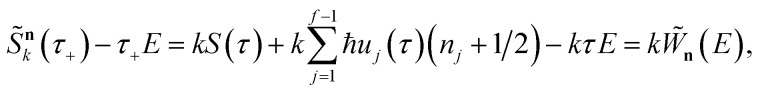
which can alternatively be rewritten in terms of the original reduced action as66
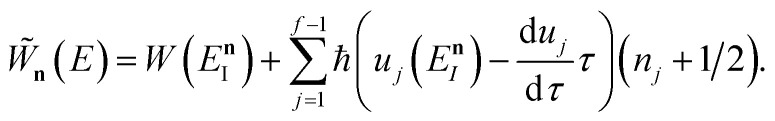


The resulting expression for the rate can then be written in the particularly simple form67
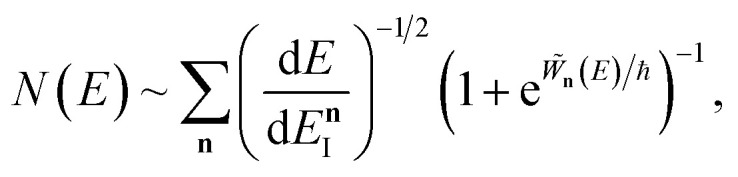
where we have used the relation between the energy and the action (eqn (22)) to combine the second derivatives of the action into a derivative of the total energy with respect to the instanton energy (an explicit expression for which can be obtained by differentiating eqn (64)).

The physical meaning of this prefactor is not entirely clear, and one may worry whether it is even generally well defined, *i.e.* if the total energy can decrease with increasing 
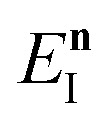
. In the following, however, we shall argue that the prefactor should not be included. We begin by noting that for separable systems, the prefactor equals one, and that when the prefactor is not close to one, this indicates that instanton frequencies are changing rapidly as a function of the instanton energy. In this case, one may expect that the starting point for our derivation, the steepest descent integration over 
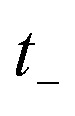
 to give eqn (39), will introduce an error as it does not account for changes to the zero-point energy along the instanton path. On this basis we propose replacing the prefactor with 1, in order to arrive at the final expression for what we term the direct-sum approach68
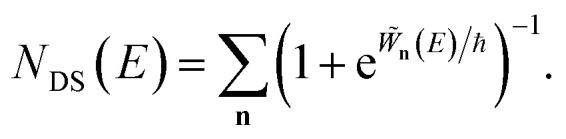


It is of interest to note that this expression can in fact be derived in an entirely different manner by following the derivation of Kryvohuz in ref. 48, with the modification of expanding the term denoted 
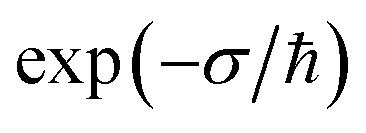
 in that paper in terms of a sum over quantum states. The derivation of Kryvohuz also explicitly attempts to account for changes in the zero-point energy along the instanton path, supporting the removal of the prefactor in this expression.

In order to complete the specification of the method, we need to define 
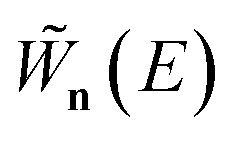
 for 
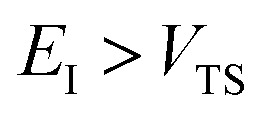
, *i.e.* for energies above the barrier where the instanton is collapsed to a point. Following the criteria used in the one-dimensional case and to ensure continuity, we suggest that69
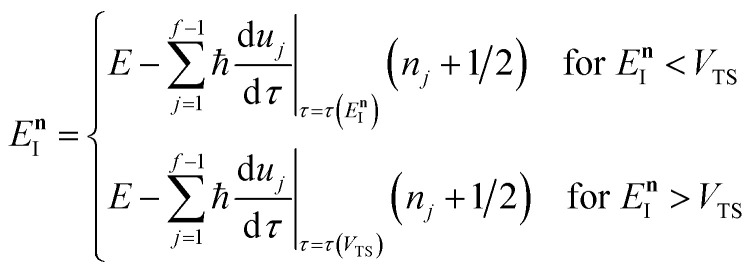
and70
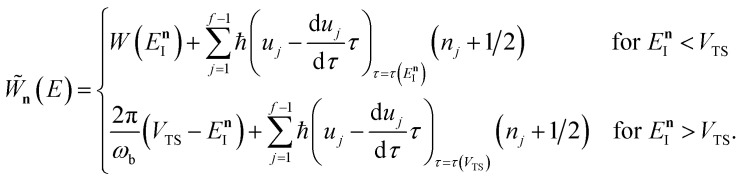


This choice is of course not unique, and whilst it is consistent with the prescription used in the one-dimensional case, neither are rigorously derived. The derivation of a rigorous instanton expression above cross-over is left for future work.

Aside from the issue mentioned above, there also exists a practical difficulty with the direct-sum method. Namely, that it requires an explicit sum over the quantum states. Unlike the microcanonical instanton of Chapman *et al.*, the explicit 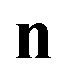 dependence of the reduced action means the cumulative reaction probability cannot be written in terms of an integral over a density of states. For a high-dimensional system, this can prove prohibitively expensive. In principle, an efficient implementation might be achieved by utilising the Wang–Landau algorithm,^49^ however this is beyond the scope of the present work.

#### 3.3.2 An improved method 2: modified density-of-states method

Here, we derive a second improved microcanonical instanton approach, with the aim of deriving a method which can be written in terms of a density of states, making it practical to apply to real systems, but which overcomes the problems seen in the DoS method and in Chapman *et al.*’s method. Note that to simplify the discussion, in the following we drop the sum over *k* and treat only the dominant term. We begin by multiplying and dividing by the instanton partition function 
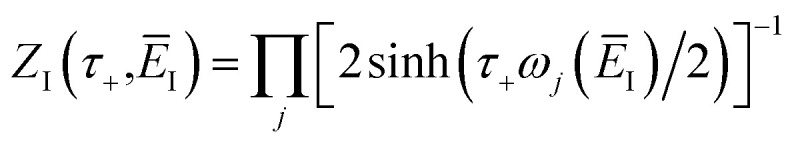
, defined at an as yet unspecified reference instanton energy 
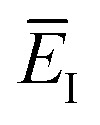
. We can write the resulting expression for the cumulative reaction probability as71
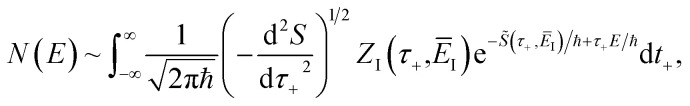
where we have defined the effective action72
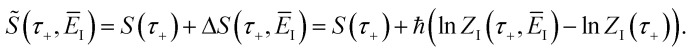


Now we can expand 
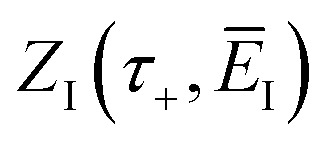
 in a sum over quantum states to give73
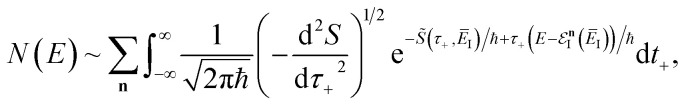
where 
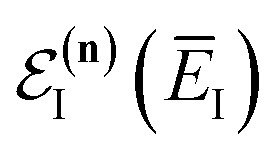
 (defined in eqn (51)) is the effective vibrational energy for an instanton path at the reference energy, 
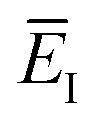
. At this stage, all we have done is multiply and divide by the same term. Note, however, that as each term in the sum over effective quantum states is now different to that in eqn (61), we will obtain a different result when integrating by steepest descent. Performing the integral by steepest descent, holding the effective vibrational energy, 
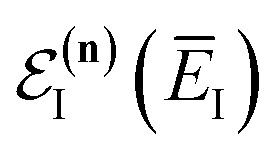
, constant, results in the condition74
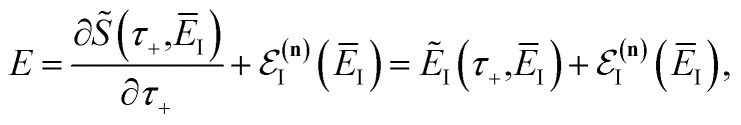
where we have defined the effective instanton energy 
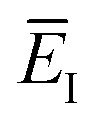
 as the derivative of the effective action. The resulting expression for the cumulative reaction probability can then be written as75
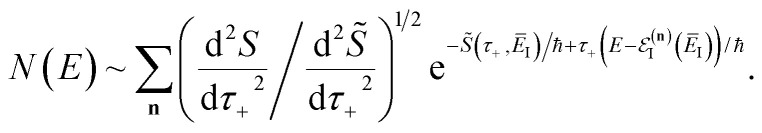


This can then further be simplified by noting that 
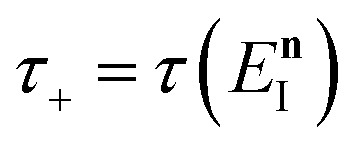
, where 
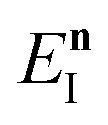
 satisfies the implicit equation76
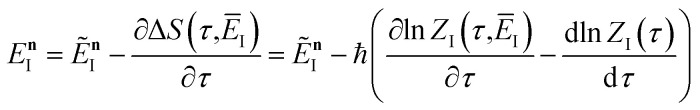
with 
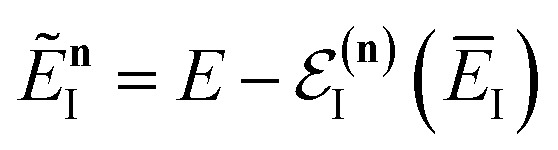
, and by defining the effective reduced action77
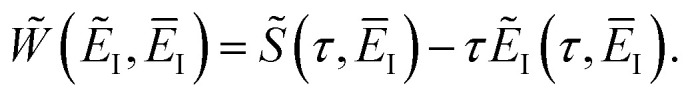


Hence, using these two relations and upon simplifying the prefactor, we can write78
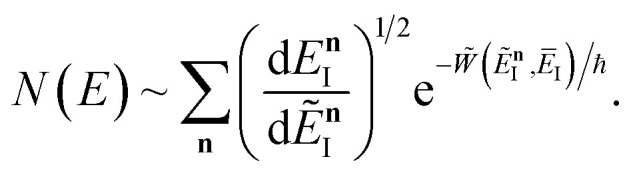


As discussed for the direct-sum approach, we suggest that it is sensible to modify this expression by removing the prefactor and replacing the WKB-like transmission probability with the parabolic barrier form to give79
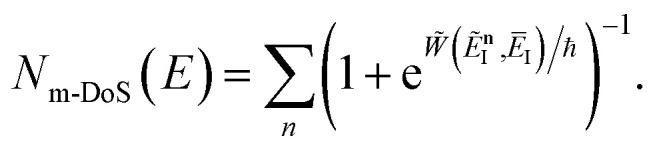


Note that this expression reduces to the separable semiclassical RRKM result (eqn (45)) in the case of a separable reaction where 
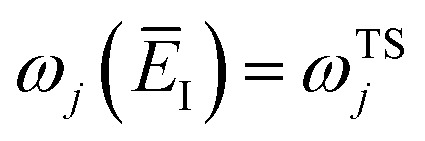
. Importantly, this expression is also straightforward to evaluate using the density-of-states approach,^44^ by writing80
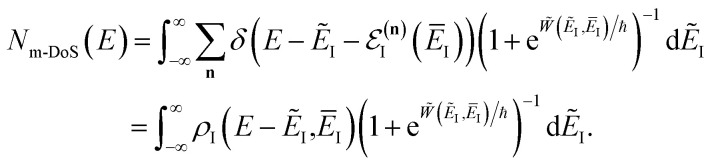


This can equivalently be transformed to give an integral over the usual instanton energy (rather than the modified instanton energy) as81
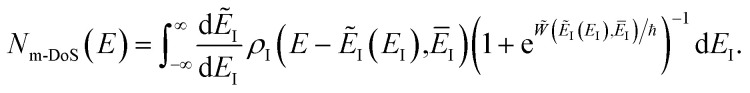


As with the direct-sum method from the previous section, there is some ambiguity as to how to define 
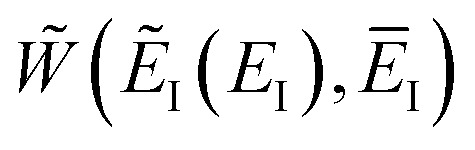
 when 
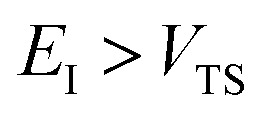
. Hence, to ensure continuity for 
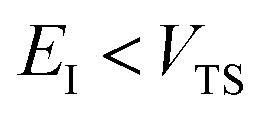
, we define82
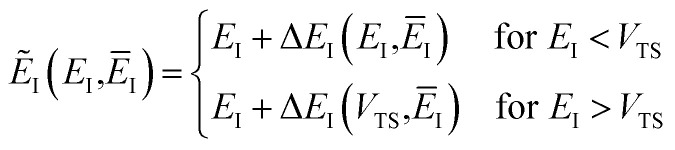
where83
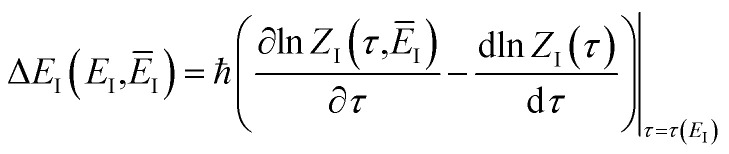
and, correspondingly,84
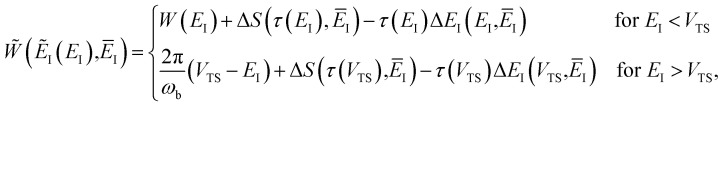
where85
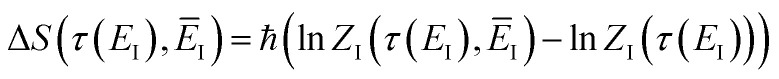


So far, we have not discussed how to define the reference instanton energy, 
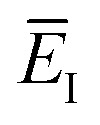
. An obvious choice is to let 
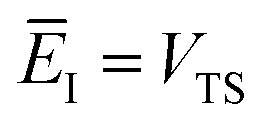
; doing so allows us to write the rate in terms of the density of states at the transition state as86
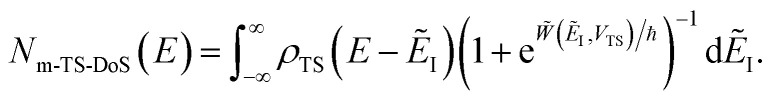


Alternatively, one could choose 
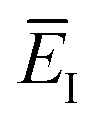
 to depend on the total energy; an obvious choice would be to define 
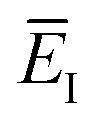
 according to the implicit equation87
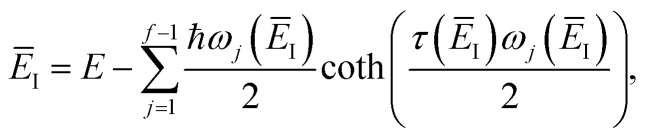
which corresponds to self-consistently choosing 
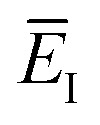
 to be the total energy minus the thermal average of the vibrational energy for the instanton density with instanton energy 
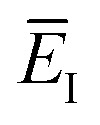
. This suggests going one step further and simply replacing 
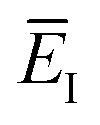
 with 
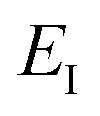
 to give88
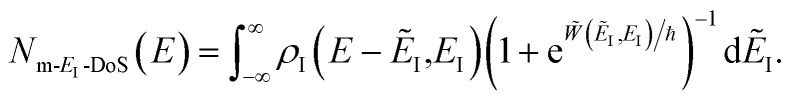


Note that, as shown in the Appendix (Section 6.2), when thermalised by integrating over energy, this can be related back to the semiclassical instanton rate.

#### 3.3.3 Application to model system

**Fig. 3:**
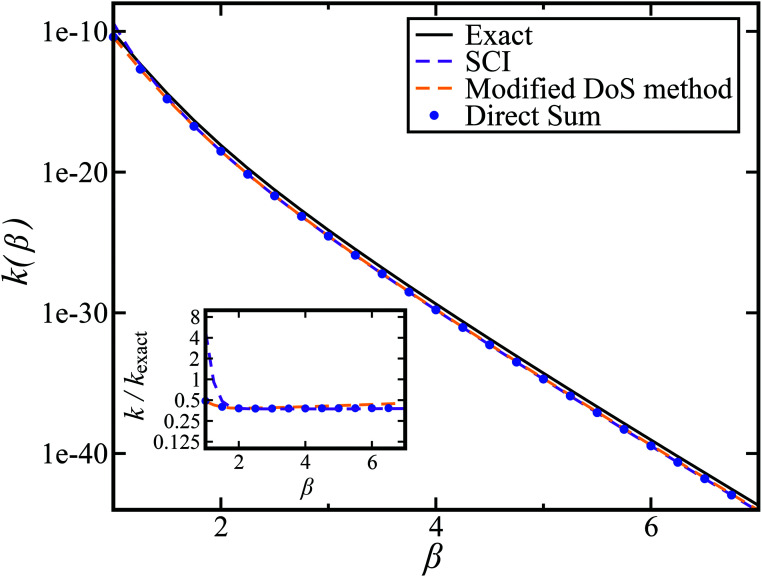
A comparison of the exact thermal rate, the semiclassical instanton rate (eqn (26)) and the thermalised microcanonical rates from the direct-sum method (eqn (68)) and the modified DoS method (eqn (88)), for model 1, with *V*_TS_ = 20, 
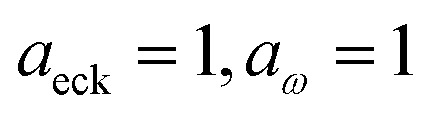
, *ω*_TS_ = 5 and *ω*_r_ = 20. The inset shows the relative error.

In order to assess how well the new microcanonical methods perform, we begin by considering the thermal rate obtained by integrating the approximate cumulative reaction probabilities over energy. Fig. 3 compares the thermal rate calculated using the modified DoS method and the direct-sum approach with both the exact rate and the semiclassical instanton rate for the same system, as in Fig. 2. For this system and the others that follow, we do not find a significant difference between each of the alternative definitions for the modified DoS methods given in eqn (86)–(88) and, hence, we show only the results for eqn (88). We expect that more generally, this will not be the case, and a thorough investigation of the optimal choice is left for later work. In contrast to the results seen in Fig. 2, both of the thermalised microcanonical instanton theories now closely follow the original thermal instanton result (SCI). Hence, we see the that for this system, the modified DoS method and the direct-sum method successfully correct the error observed in the original DoS method and in Chapman *et al.*’s method. These new microcanonical methods are, however, still not more accurate than the original thermal instanton, with a comparable error of around −60% seen across a wide range of temperatures. The only exception to this is close to the cross-over temperature, where the thermalised microcanonical methods perform much better than the original thermal instanton theory.

**Fig. 4:**
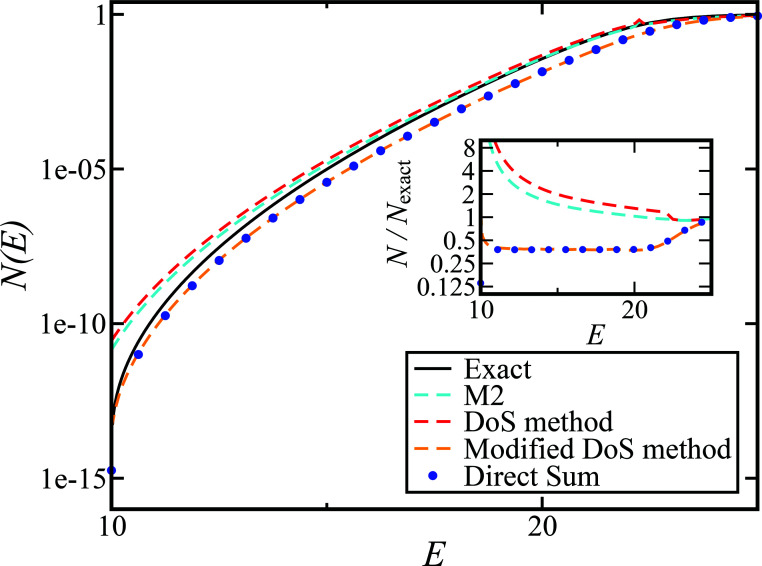
A comparison of the exact cumulative reaction probability, with the microcanonical instant result calculated using Chapman *et al.*’s method (eqn (49)), the DoS method (eqn (52)), the direct-sum method (eqn (68)) and the modified DoS method (eqn (88)) for model 1, with *V*_TS_ = 20, 
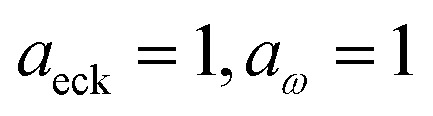
, *ω*_TS_ = 5 and *ω*_r_ = 20. The inset shows the relative error.

To understand the origin of this error in more detail, we can examine the cumulative reaction probability directly. Fig. 4 compares the exact cumulative reaction probability for the same system, with approximate results calculated using each of the four microcanonical instanton methods considered so far. We can see that the errors observed in the thermal rate for Chapman *et al.*’s method (M2) and the DoS method are primarily caused by error at the lowest energies, while close to the threshold energy, both theories agree well with the exact result. The reasons for this close agreement are not obvious and may be the result of error cancellation, particularly in light of the importance of anharmonic effects, which are discussed later. In contrast, the direct-sum method and the modified DoS method are both most accurate at low energies, with a noticeable error relative to the exact result at intermediate values of energy for this system. However, the close agreement between the two methods, in combination with the close agreement between the thermalised rates and the semiclassical instanton, indicates that this is an error inherent in the semiclassical instanton method itself.

### 3.4 Anharmonic correction

One explanation for the remaining discrepancy between the microcanonical instanton and the exact cumulative reaction probabilities is that anharmonic fluctuations around the instanton path are important. Whilst the instanton partition function captures the change in frequency along the instanton path, as is clear from its functional form it still inherently assumes that the fluctuations about the path are harmonic. In order to test the hypothesis that it is this harmonic assumption which leads to the error seen in Fig. 4, we therefore need to include anharmonic fluctuations around the instanton path. To achieve this, it is helpful to consider the Im-F formulation of the instanton rate.

Within the Im-F formalism, the rate is postulated to be given approximately by^16^89
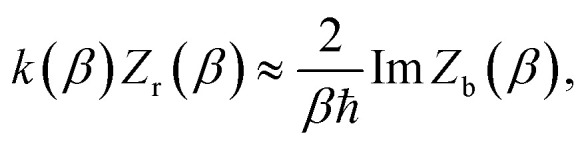
where 
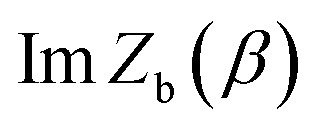
 is the “imaginary part of the barrier partition function”. Implicit in this is the idea that the unstable mode at the barrier is integrated by steepest descent using analytic continuation such that the otherwise divergent integral gives an imaginary result. To derive the instanton rate in the Im-F framework, one can begin by defining the free-energy 
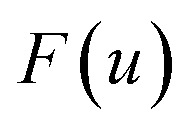
 along the unstable coordinate according to^13^90
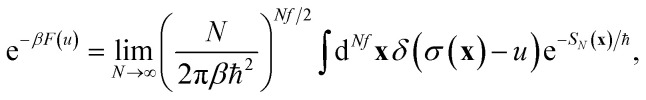
where 
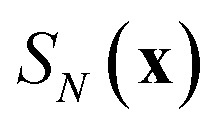
 is the ring-polymer action given by eqn (21) and 
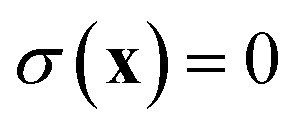
 is a dividing surface which separates the reactants from products in the ring-polymer space and which passes through the transition-state/instanton geometry on the ring-polymer potential-energy surface, 
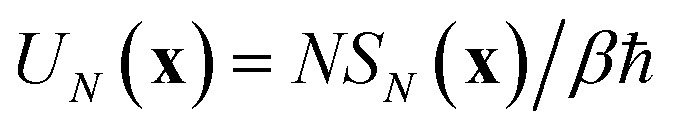
, and maximises the free energy 
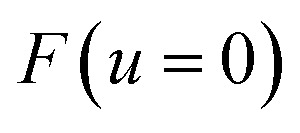
. Integrating first over the unstable *u* coordinate using the Im-F prescription (including a factor of a half since the analytic continuation only integrates over half the peak)^50^ gives91
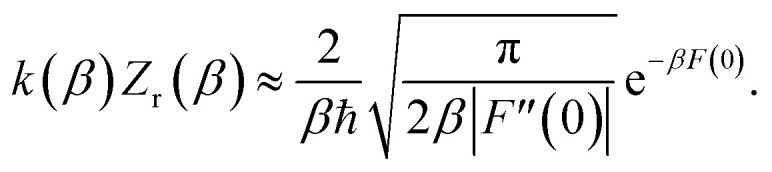


If the free-energy and its second derivative are then also evaluated by steepest descent 
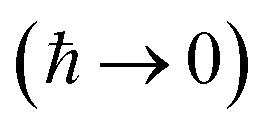
, then one recovers the thermal instanton rate92
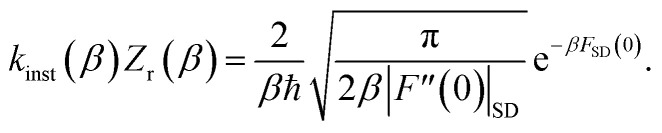


The resulting formula is formally equivalent to those given above (eqn (26)).^34,42^

To test the importance of anharmonic fluctuations, we therefore propose the following simple anharmonic correction factor,93
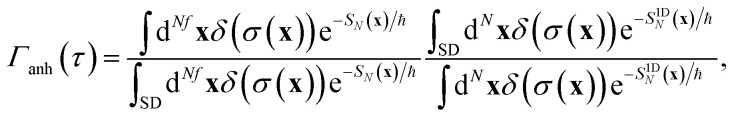
where in order to remove the contribution from fluctuations parallel to the instanton path itself, we have divided by the ratio of the steepest descent and full path integral for the one-dimensional Eckart-barrier part of the model. For more complex problems, the equivalent effect could be achieved by creating an effective one-dimensional model defined by the potential along the instanton path (which can be continued beyond the instanton using real time classical trajectories initiated at the turning points of the trajectory, or the minimum-energy path down to the well bottoms). The anharmonic correction factor is then included in the microcanonical rates by replacing Δ*S* from eqn (85) with94
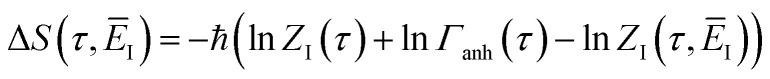
and, correspondingly, 
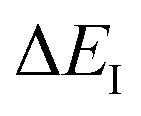
 (from eqn (83)) with 
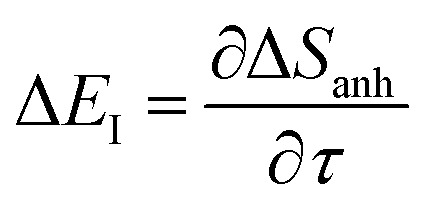
. Note that for the model systems considered here, whilst the 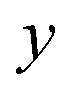 coordinate is harmonic for a fixed value of 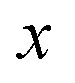, the 
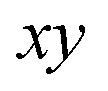
 coupling can lead to anharmonic fluctuations. It is these fluctuations which are included in the anharmonic correction factor.

**Fig. 5:**
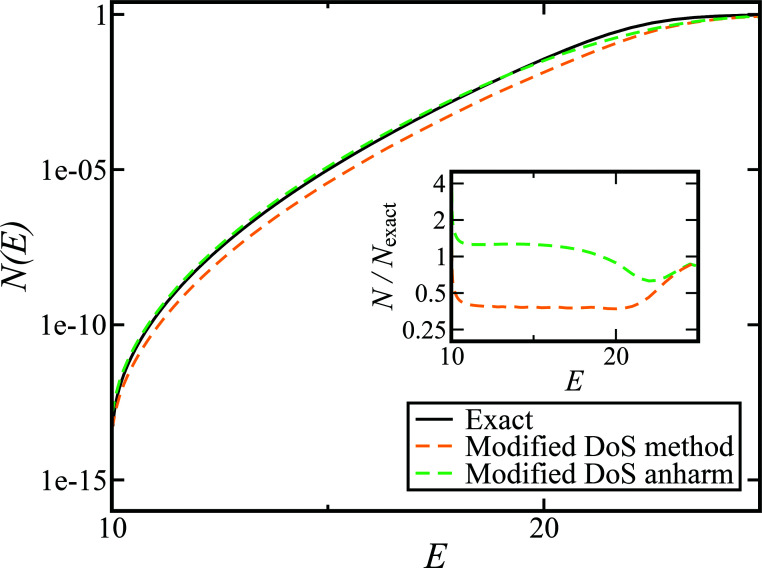
A comparison of the exact cumulative reaction probability for the modified DoS method (eqn (88)) and the anharmonic-corrected modified DoS method (eqn (94)) for model 1, with *V*_TS_ = 20, 
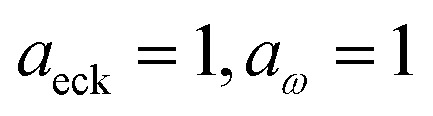
, *ω*_TS_ = 5 and *ω*_r_ = 20. The inset shows the relative error.

Fig. 5 shows the results of including the anharmonic correction for the same model as in Fig. 4. The resulting cumulative reaction probability, calculated using the modified DoS method, now agrees very well with the exact results for a wide range of energies. This confirms the hypothesis that the main cause of the discrepancy between the instanton results and the exact rate is indeed the neglect of anharmonic fluctuations around the instanton path. We note that the anharmonic correction used here is not necessarily the optimal choice for a real molecular system, and one could instead imagine using correction factors based on the closely related ring-polymer transition state theory or on the quantum instanton approach,^46,51–55^ but this aspect is left for future work.

**Fig. 6:**
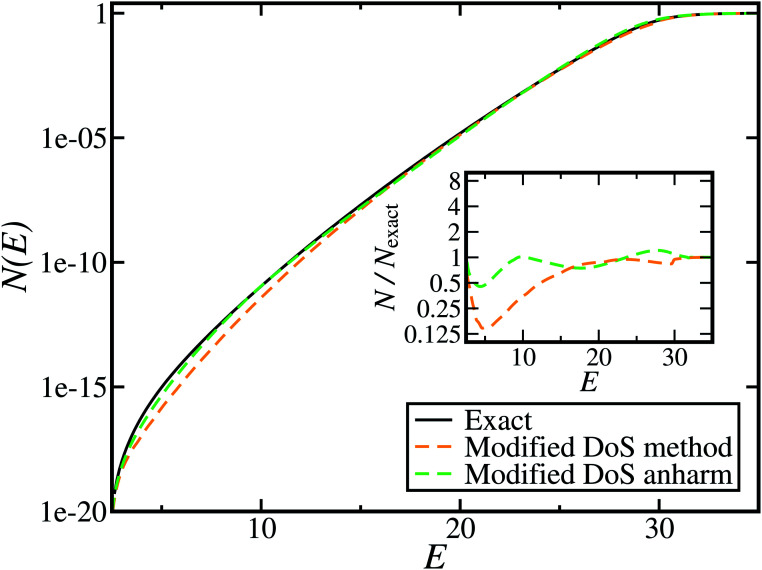
A comparison of the exact cumulative reaction probability from the modified DoS method (eqn (88)) and the anharmonic-corrected modified DoS method (eqn (94)) for model 2, with *V*_TS_ = 20, *a*_eck_ = 1, 
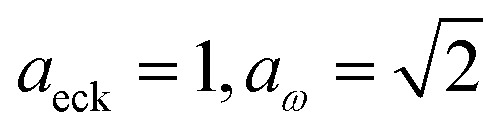
, *ω*_TS_ = 20 and *ω*_r_ = 5. The inset shows the relative error.

Although the anharmonic-corrected modified DoS method agrees well with the exact result at most energies considered, there is still a small difference close to the threshold energy (*i.e.* where the tunnelling becomes important) for this system. The *ad hoc* nature of the instanton correction in the cross-over region is likely the cause of this discrepancy. However, we note that the difference here is particularly pronounced, as the frequency of the *y* coordinate changes rapidly near the top of the barrier. This rapid change means that 
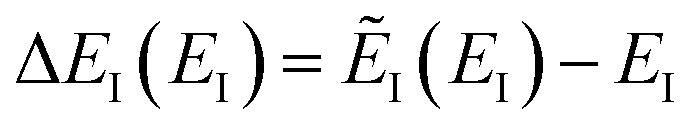
 has a non-zero value at 
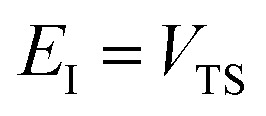
. For systems where the frequency changes more slowly close to the top of the barrier, one would therefore not expect to see such a discrepancy. This is illustrated in Fig. 6, where both the anharmonic-corrected modified DoS methods and the uncorrected method are applied to model 2 with 
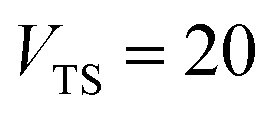
, 
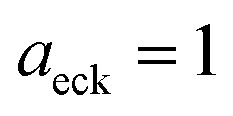
, 
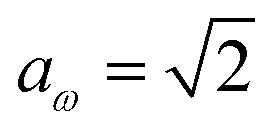
, 
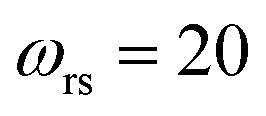
 and 
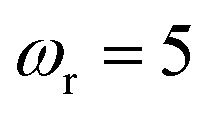
. Here, the frequency of the 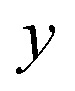 coordinate is approximately constant in the vicinity of the barrier top and, correspondingly, the anharmonic-corrected method now agrees well with the exact results at all energies considered. This situation may be expected to be more typical of real systems, where the length scale for changes to the orthogonal frequencies may be comparable to the change in the curvature along the unstable mode. This analysis seems to imply that such systems require us to go beyond the steepest descent approximation. However, there is an alternative possibility that by rigorously deriving the contribution from terms which involve multiple instanton orbits, this error could be reduced, and this therefore presents an interesting topic for further research.

## 4 Application to a unimolecular reaction

One of the things that makes thermal semiclassical instanton theory so successful is that it is straightforward to apply to real molecular systems. The typical workflow for the calculation of thermal instanton rates consists of first finding the classical transition state corresponding to the high-temperature limit of the instanton, and then optimising (ring-polymer discretised) instantons at successively lower temperatures, using the previous instanton as a starting point for the optimisation.^34^ The calculation of microcanonical instanton rates using either the density-of-states or modified density-of-states instanton methods only involves some additional post-processing on top of this usual workflow. To calculate the microcanonical cumulative reaction probabilities, one simply takes the data calculated for a thermal instanton at a set of temperatures and then generates a series of spline fits for the relevant quantities, from which the microcanonical result at a desired energy can then be calculated.^44^

In order to demonstrate the applicability of the microcanonical instanton approach, we apply it here to a well studied molecular system, the unimolecular dissociation of H_2_CO to H_2_ + CO.^56–67^ This system has been of continued scientific interest over at least the last 50 years due to its importance in combustion, interstellar and atmospheric chemistry, and more recently as it was one of the first systems in which roaming was proposed to play a role (although here we focus on the tunnelling regime where roaming trajectories do not play a role).^61–64^ We also note that it is of historical interest from the perspective of microcanonical rate theory as it was the system for which Miller’s popular Eckart tunnelling correction to the RRKM rate was originally proposed and applied.^17^ This system also has the advantage that considerable effort has already gone into developing accurate potential-energy surfaces (PES) for studying the roaming dynamics in this system.^62,66^ Hence, for the following, we use the 2017 PES developed by Wang, Houston and Bowman in ref. 66.

### 4.1 Practical implementation for molecular systems

Before we apply the modified density-of-states method to this reaction, we outline a few implementational details relevant to the application to molecular systems. Firstly, we must consider the generalisation to include rotations. This is rather straightforward, since the moments of inertia typically do not change significantly as a function of the instanton energy. Hence, in the following, we simply replace the instanton density of states in eqn (88) with the density of states for the combined rotations and vibrations of the instanton, given formally for a non-linear system as95
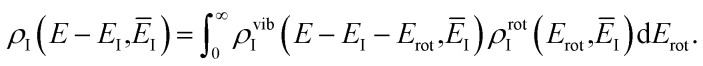


Note that in a case where the moment of inertia does change significantly as a function of 
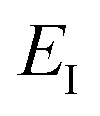
, then one can also include a factor of 
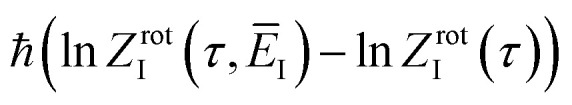
 in the definition of 
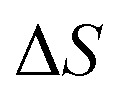
. The vibrational density of states is defined as before, 
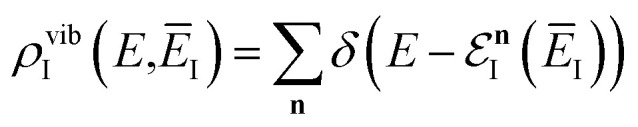
 and, consistent with standard thermal semiclassical instanton theory,^34^ we use the classical rotational density of states, given formally as96
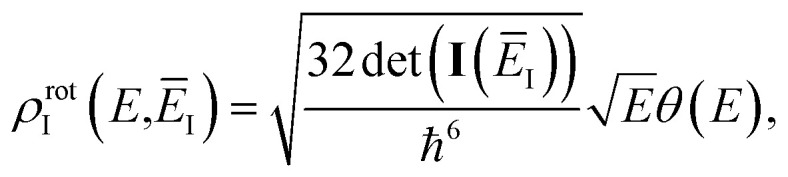
where **I**(*E*_I_) is the moment of inertia tensor for the instanton with instanton energy *E*_I_. Note that here we have only considered energy-resolved microcanonical rates. It would, however, be straightforward to generalise the present theory to give microcanonical rates that are also resolved by total angular momentum, or to go beyond the simple model of separable classical rotations.^68^

Practically, however the density of states is not calculated using these relations, and instead is calculated using the stationary-phase approximation to the inverse Laplace transform (SPA-ILT), as proposed in ref. 44. This extra approximation is straightforward to compute in closed form without the need to integrate over the rotational energy or sum over the vibrational states, and typically leads to an error of less than 10%. Finally we note that to convert from the cumulative reaction probability, 
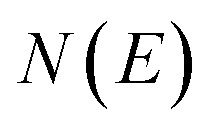
, to the microcanonical rate constant, 
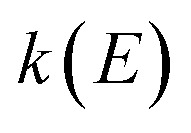
, one must divide by the reactant density of states at energy 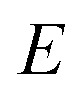; in keeping with the rest of the present work, the reactant density of states is calculated using a harmonic-oscillator and rigid-rotor approximation in combination with the SPA-ILT method.

### 4.2 Application to H_2_CO → H_2_ + CO dissociation

In addition to the modified DoS method, for comparison we also calculate the microcanonical rate using the commonly-used separable Eckart tunnelling correction proposed by Miller in ref. 17. In this theory, one fits an asymmetric Eckart barrier to reproduce the imaginary barrier frequency and the barrier heights relative to the reactant and product potential energies, and then uses the corresponding analytic one-dimensional barrier transmission probability to modify RRKM, as in eqn (11). In keeping with the approach used for the microcanonical instanton theories, we also use the SPA-ILT approximation to calculate the density of states.

**Fig. 7:**
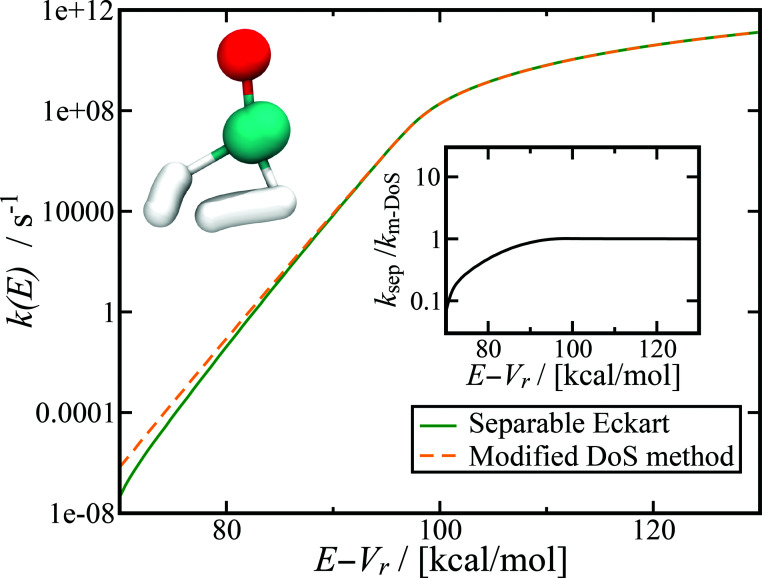
Microcanonical reaction rate constant for the H_2_CO → H_2_ + CO dissociation. Note that for this system, the modified DoS and original DoS methods are identical to graphical accuracy and, hence, only the modified DoS results are shown. The inset shows the ratio of the separable approximation relative to the modified DoS result, highlighting the breakdown of the separable approximation at low energies. The top left of the figure shows an example of the instanton tunneling path.

Fig. 7 shows the microcanonical rate constant for the H_2_CO → H_2_ + CO dissociation as a function of energy relative to the potential-energy minimum for the H_2_CO molecule for both the modified DoS method and the separable Eckart-corrected RRKM theory. Over a wide range of energies, the separable Eckart correction to the RRKM and the microcanonical DoS instanton agree closely. However, below *E* − *V*_r_ ≈ 85 kcal mol^−1^, the separable Eckart result begins to deviate significantly from the instanton result, with an error of more than a factor of 2 for energies below *E* − *V*_r_ = 80 kcal mol^−1^ and more than a factor of 10 at *E* − *V*_r_ = 70 kcal mol^−1^. This error can be primarily attributed to the failure of the separable approximation, which means that the change to the density of states for the deep-tunnelling instanton paths is not captured. We note that these energies are lower than those that can be accessed directly *via* the photoexcitation process, where the initial excitation to S_1_ is followed by internal conversion to 
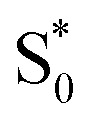
. The lowest allowed transition for this process is around 80.9 kcal mol^−1^ (ref. 65) (note that the zero-point energy of H_2_CO calculated using the present PES is ≈16.4 kcal mol^−1^, implying that the lowest accessible *E* − *V*_r_*via* this photoexcitation pathway is ∼97 kcal mol^−1^). Although for this system the region of energy where non-separable effects are important is not directly accessible by this process, there is no reason to believe that this will be true generally. In any case, the results seen here serve to illustrate the utility of microcanonical instanton theory as a way of going beyond simple one-dimensional tunnelling corrections. Furthermore, lower energies are of course accessible for this system *via* collisional deactivation of the excited H_2_CO and, hence, this region is still of interest in fully characterising the dynamics of photoexcited H_2_CO.

Although this system is sufficiently non-separable that the Eckart tunnelling correction to RRKM fails at low energy, it is not sufficiently non-separable to lead to a significant deviation of the modified and original density-of-states instanton methods. This indicates that for this system, the instanton frequencies change sufficiently slowly with instanton energy that the corrections to the action and instanton energy do not deviate significantly from the unmodified result. We do not expect this to be true in general, as previous work has demonstrated that the stability parameters can be strongly dependent on temperature.^69,70^ It will thus be an interesting area of future research to find a system for which the original DoS method breaks down, as well as to test the current method against experimental results.

## 5 Conclusion

To accurately describe reactions at low energies and low temperatures, methods which can capture the effects of quantum-mechanical tunnelling are required. For thermal reactions, instanton theory is now a well-established method for calculating reaction rates. Because instanton theory locates the optimal semiclassical tunnelling path in full dimensionality, it is especially useful in systems where effects such as corner cutting mean that simple one-dimensional tunnelling corrections along the minimum-energy path break down. The task of calculating microcanonical reaction rates is a fundamentally harder problem. This is in part because of the ill-conditioned nature of the inverse Laplace transform, which relates the thermal rate to its microcanonical counterpart. Additionally, whilst the semiclassical approximation 
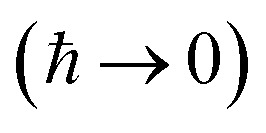
 of instanton theory typically works well in the time domain, it can lead to significant errors when taking only the leading term in the energy domain, as seen in the original microcanonical instanton theory developed by Miller.

The failure of Miller’s original microcanonical theory led Chapman, Garrett and Miller to suggest an *ad hoc* fix to the theory in order to recover the one-dimensional tunnelling correction to RRKM theory for separable systems. Here, we have demonstrated that this fix does not always lead to a result which can accurately recover the thermal semiclassical instanton, in particular for strongly non-separable systems. By reconsidering the derivation from first principles, we have suggested an improved method, the modified DoS method, that still recovers the separable tunnelling correction to RRKM in the appropriate limit, but can also be applied to strongly non-separable systems. We have illustrated the accuracy of this method by comparing it to both exact results as well as thermal instanton results for a series of model problems. Whilst the agreement between the thermal semiclassical instanton and the thermalised modified DoS method is good, there remain a number of interesting areas for further theoretical work. In particular, the development of anharmonic corrections to the instanton rate, the identification of the optimal reference instanton energy for the density of states, and also the rigorous derivation of the multiple bounce contributions to the rate in the cross-over region from deep to shallow tunnelling. We also note that the present work has only considered systems for which the Born–Oppenheimer approximation is valid in the vicinity of the reaction barrier. However, it would be interesting to consider generalising the ideas discussed here to treat electronically nonadiabatic systems, in combination with nonadiabatic instanton methods.^8,71–74^

We have demonstrated the applicability of the improved method to molecular systems in full dimensionality with an application to the unimolecular dissociation of H_2_CO. Our results illustrate the need to go beyond simple one-dimensional tunnelling corrections, which were seen to fail at low energies. For this system, these non-separable tunnelling effects are below the energies directly accessible experimentally *via* the standard photoexcitation pathway. This suggests an interesting area of future work to find microcanonical systems where non-separable quantum tunnelling effects can be directly experimentally probed.

## 6 Appendix

### 6.1 Stability parameters

The stability parameters for a classical trajectory, 
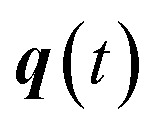
 can be calculated from the eigenvalues of the monodromy matrix (also known as the stability matrix) 
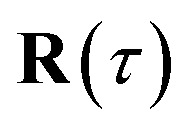
. Formally, the monodromy matrix for the imaginary time trajectory 
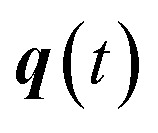
 can be written in terms of the time-ordered exponential of the “force constant matrix”^11^97
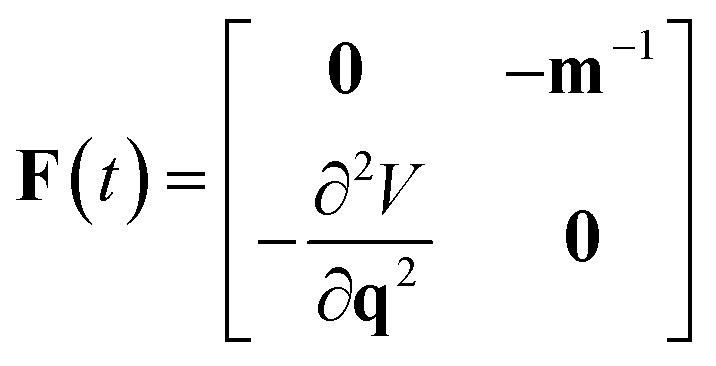
as98
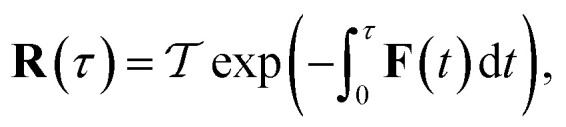
where 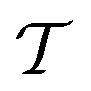 is the time-ordering operator. The eigenvalues of the monodromy matrix occur in 
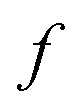
 pairs of the form 
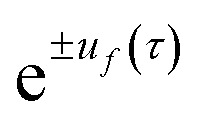
, where 
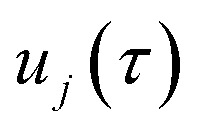
 are the stability parameters of the orbit. For a model potential containing no translational or rotational symmetry, there is one zero stability parameter corresponding to the cyclic symmetry of the path, and for a gas-phase non-linear molecular system, there will be 7 zero stability parameters, corresponding to the 3 translational and 3 rotational as well as the 1 cyclic degree of freedom.

Unfortunately, as the stability parameters scale with the length of the periodic orbit 
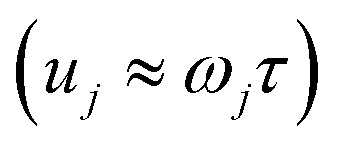
, for even moderately low temperatures the eigenvalues can become very large. This can become a numerical issue if the difference between the smallest and largest eigenvalue of the monodromy matrix is close to the precision used to store the matrix. In the past, this has presented an issue for the numerical calculation of stability parameters.^70,75^ However, arbitrary-precision linear-algebra routines are now widely available (*e.g.* in the python package mpmath^76^ and FLINT^77^) and highly optimised, such that the computation of stability parameters is straightforward even for systems at very low temperatures. Optionally, to minimise the number of operations performed using arbitrary-precision arithmetic, one can split the monodromy matrix into *M* parts,99
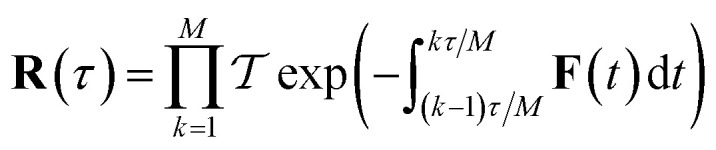
such that each part can still be stored accurately at double precision. Each of the 
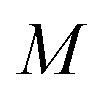
 shorter time-ordered exponentials can then be calculated trivially from the discretised instanton path, before being combined together using an arbitrary-precision software package and diagonalised.

### 6.2 Thermalising modified DoS method by steepest descent

We show here how the modified DoS method is related to the thermal semiclassical instanton method. We begin by thermalising eqn (88) to give100
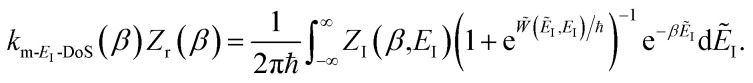
which in the deep-tunnelling regime can be approximated using just the leading WKB-like term in the expansion of the transmission probability to give101
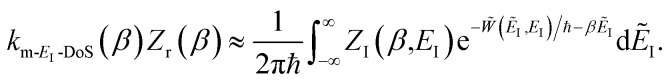


Transforming from the modified instanton energy to the usual instanton energy gives102
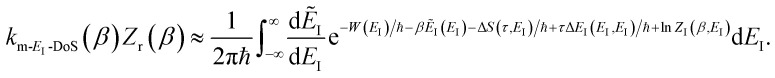


Now, when integrating over *E*_I_ by steepest descent, the resulting steepest descent condition is given by103
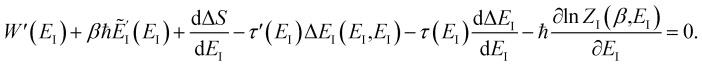


This is solved by the condition 
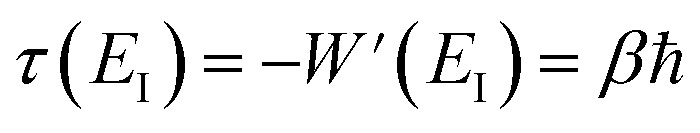
, which can be seen by noting that under this condition104
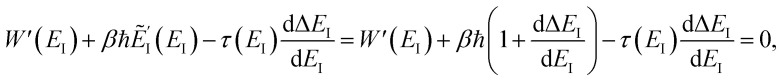
and105
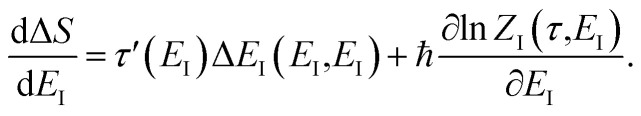


Hence, integrating by steepest descent recovers the usual thermal semiclassical instanton theory up to a difference in the prefactor.

## Conflicts of interest

There are no conflicts to declare.

## Supplementary Material
